# Additively Manufactured Bionic Cellular Metamaterials with Controllable Thermal Conductivity—Mathematical Models and Experimental Research

**DOI:** 10.3390/ma19142992

**Published:** 2026-07-10

**Authors:** Beata Anwajler

**Affiliations:** Faculty of Mechanical and Power Engineering, Wroclaw University of Science and Technology, 27 Wybrzeze Wyspianskiego Street, 50-370 Wroclaw, Poland; beata.anwajler@pwr.edu.pl

**Keywords:** thermal metamaterials, open-cell lattices, additive manufacturing, thermal conductivity, architected cellular materials, homogenization theory, heat transfer, lattice structures

## Abstract

**Highlights:**

- A topology-based mathematical model for open-cell thermal metamaterials was developed.
- Thermal conductivity depends on topology rather than porosity alone.
- BCC and Diamond lattices provided the lowest effective thermal conductivity.
- Model predictions agreed with experiments with an average error of approximately 5.6%.
- The proposed approach enables topology-driven thermal metamaterial design.

**Abstract:**

Bio-inspired cellular metamaterials manufactured using additive manufacturing technologies provide a promising route for controlling thermal transport properties through architecture rather than through the intrinsic properties of the constituent material. This study investigates steady-state heat transfer in open-cell lattice structures comprising 20 different lattice metamaterial specimens representing various classes of cellular architecture. These include Kelvin, auxetic, BCCZ, BCC, cube, Z-cuboctahedron, diamond, FCC, FBCCXYZ, FCCZ, FBCC, G7, isostructure, octahedron, octet structure, rhombohedral dodecahedron, truncated cuboctahedron and truncated cube, all of which are made from polymer materials. The investigated architectures were inspired by functional principles observed in natural cellular systems, including cancellous bone, wood, coral skeletons, and other biological porous materials, where efficient transport processes are achieved through optimized material distribution and interconnected cellular networks. A theoretical model combining conduction through the lattice skeleton, radiative heat transfer within pores and potential convective contributions was developed using homogenization theory and representative volume element analysis. The experiment confirmed the main hypothesis of this study as described by the mathematical model. Experimental validation also confirmed that the homogenization model correctly predicts the thermal conductivity of open-cell lattice structures in highly porous materials with a porosity of around 0.95. The results demonstrate the potential of biomimetic cellular design for the development of lightweight thermal-management materials with programmable thermal transport properties.

## 1. Introduction

Due to the continuously increasing costs of energy and raw materials, the enhancement and optimization of heat transfer processes have become critical challenges across numerous engineering disciplines [1]. Consequently, there is a growing demand for highly efficient systems and devices capable of significantly reducing both structural dimensions and operational costs while maintaining high thermal performance [2]. Heat transfer constitutes one of the fundamental physical phenomena governing the operation of technical systems in environmental engineering, mining, and the energy sector. Effective thermal management directly affects the efficiency of energy conversion processes, the operational safety of industrial installations, the durability and reliability of construction materials, and the overall environmental impact of engineering systems. At the macroscopic scale, these issues are associated with the thermal insulation of building envelopes, thermal energy storage, heat losses in transmission systems, and the thermal stability of infrastructure facilities [3,4]. At the micro- and mesoscale, they involve the design and engineering of materials and structures with precisely tailored thermal transport properties.

The traditional approach to controlling thermal conductivity is primarily based on material selection, in which thermal conductivity is treated as an intrinsic material property resulting from its atomic structure and chemical composition. Within this framework, the modification of thermal properties is generally limited to the use of materials exhibiting either high or low thermal conductivity, or to the fabrication of composite materials, where the effective thermal behavior depends on the volume fraction and spatial distribution of the constituent phases. Although this approach has proven effective in numerous engineering applications, it reveals significant limitations in systems requiring the simultaneous fulfillment of conflicting design criteria, such as low density, high mechanical strength, controlled thermal anisotropy, and adaptability to variable operating conditions [5,6]. In response to these challenges, recent years have witnessed rapid advances in the development of bionic materials and metamaterials, in which the governing role is played not only by the intrinsic properties of the constituent material, but primarily by the deliberately designed geometry and topology of the internal architecture [7,8,9]. Within this paradigm, thermal conductivity is no longer regarded solely as a material property, but rather as a parameter that can be engineered and tailored through the controlled design of the internal structure.

### 1.1. Introduction to Thermal Metamaterials

Metamaterials are defined as artificially engineered materials whose effective physical properties arise primarily from the architecture of their internal structure rather than from the intrinsic properties of the base material itself [3,4]. The adaptation of the metamaterial concept to heat-transfer phenomena has led to the emergence of thermal metamaterials, enabling the manipulation and control of heat flux pathways [10,11,12,13]. It should be emphasized, however, that heat transfer in most engineering applications is predominantly diffusive in nature [4,14].

Thermal metamaterials enable functionalities that are unattainable in conventional natural materials, including directional thermal conductivity, heat-flux concentration or dissipation, and even thermal analogues of classical devices known from other branches of physics, such as cloaks, rotators, and thermal concentrators. A key feature of thermal metamaterials is their internal architecture, typically realized as periodic or quasi-periodic structures with a characteristic geometric scale significantly larger than the atomic scale but smaller than the overall dimensions of the engineered component [10,11]. This multiscale character enables the application of classical engineering approaches for describing heat transfer, such as homogenization theory and heterogeneous-medium models, while simultaneously providing effective properties that exceed the limitations of conventional materials.

In the context of environmental engineering and energy systems, thermal metamaterials open new design possibilities by enabling the reduction in energy losses, localized control of heat flow, and the integration of structural and thermal-insulation functions within a single material system. Particularly promising among these materials are cellular structures, which, due to their geometry, combine low density with the capability for precise tailoring of effective thermal properties.

### 1.2. Thermal Conductivity Anisotropy in Cellular Metamaterials

Many architected lattice materials may exhibit anisotropic thermal transport because their strut connectivity and heat-transfer pathways are direction-dependent [15,16,17]. This effect is particularly relevant for BCC-, FCC-, octet-, tetrahedron-, and TPMS-based architectures, where the effective thermal response may vary with the orientation of the applied temperature gradient [16,18,19].

In the present study, however, experimental validation was performed under one-dimensional vertical heat-flow conditions. Therefore, the effective thermal conductivity reported in this work should be interpreted as a directional effective property corresponding to the investigated top-to-bottom or bottom-to-top configuration. Under these conditions, the scalar effective thermal conductivity assumption provides a suitable first-order approximation for comparing topology-dependent thermal insulation performance [20,21]. A complete tensor-based description of anisotropic thermal conduction, including the general conductivity tensor, orthotropic simplifications, anisotropy ratios, and rigid coordinate rotation, is provided in the Supplementary Materials, Section S1.

### 1.3. Cellular Structures as Carriers of Metamaterial Functions

Over recent decades, research on heat flow and heat transfer in porous and cellular media has gained significant importance due to its broad range of scientific and engineering applications [12]. Biological structures, owing to their exceptional multifunctional properties, frequently serve as inspiration for the development of high-performance engineered systems [13,22]. Over millions of years of evolution, living organisms have developed structures with unique characteristics that continue to provide new concepts for the design of efficient engineering materials and architectures [13,23]. Bionics offers the possibility of solving scientific and technological problems by learning from highly optimized natural structures and materials [24]. Consequently, the artificial fabrication of biologically inspired structures and multifunctional materials with enhanced performance has become a subject of considerable interest [25]. In the development of cellular structures, researchers are primarily inspired by natural systems such as bone architectures, butterfly wings, Norway spruce, mantis shrimp exoskeletons, beetles, and diving spiders [13,23].

Lightweight structural systems are increasingly important in numerous engineering sectors, including aerospace, transportation, nuclear engineering, and civil infrastructure. As a result, a wide variety of advanced lightweight materials and architectures have been developed in recent years, including columnar structures [26,27], layered systems [28,29], honeycomb materials [30], foams [23], architected cellular materials [31], and lattice structures with smooth cellular geometries [32]. Various approaches have been proposed for utilizing porous media to enhance heat-transfer performance [13]. One of the most widely applied techniques involves the partial or complete filling of heat-exchanger channels with porous materials. Such an approach increases the effective contact surface area between the coolant and the heat exchanger, thereby significantly improving heat-transfer efficiency. Research in this field includes experimental investigations of different porous materials, optimization of channel geometries, and analyses of the influence of operating parameters on thermal performance [33,34,35,36,37,38,39] Consequently, these studies may contribute to the development of more efficient and energy-saving heat-transfer systems, which is highly relevant both for industrial applications and sustainable engineering.

Cellular structures, occurring in the form of foams, lattices, and regular cellular architectures, have long been employed in engineering as lightweight materials with favorable strength-to-weight ratios. Their mechanical, acoustic, and thermal properties are strongly governed by cell geometry, porosity level, and the connectivity of the load-bearing elements [5,40]. In contrast to homogeneous materials, heat transfer in cellular structures occurs through complex coupled mechanisms, including conduction through the solid skeleton, possible contributions from the gas phase or pore-filling materials, and contact effects at cell interfaces.

From the perspective of thermal metamaterials, a particularly important aspect is that modifications in cellular topology may lead to substantial changes in the effective thermal conductivity, independently of the intrinsic properties of the base material. Properly designed cellular architectures may exhibit strong thermal anisotropy, directional heat-transport pathways, or the ability to suppress heat flux within selected regions [5]. Consequently, cellular structures constitute a natural candidate for the realization of thermal metamaterials with functionalities tailored to specific engineering applications.

### 1.4. Biomimetic Design Principles of Cellular Thermal Metamaterials

Biological materials frequently achieve exceptional multifunctional performance through optimized structural organization rather than through the intrinsic properties of their constituent materials alone. Natural cellular systems such as cancellous bone, wood, bamboo, coral skeletons, radiolarians, diatoms, and marine sponge structures combine low density with efficient transport of mechanical loads, fluids, and thermal energy through highly interconnected porous networks and hierarchical architectures [41,42].

A common characteristic of these biological systems is the presence of cellular architectures that maximize functionality while minimizing material consumption. Such structures typically exhibit large surface-to-volume ratios, optimized transport pathways, hierarchical organization across multiple length scales, and topology-dependent anisotropic properties [19,41].

**Figure 1:**
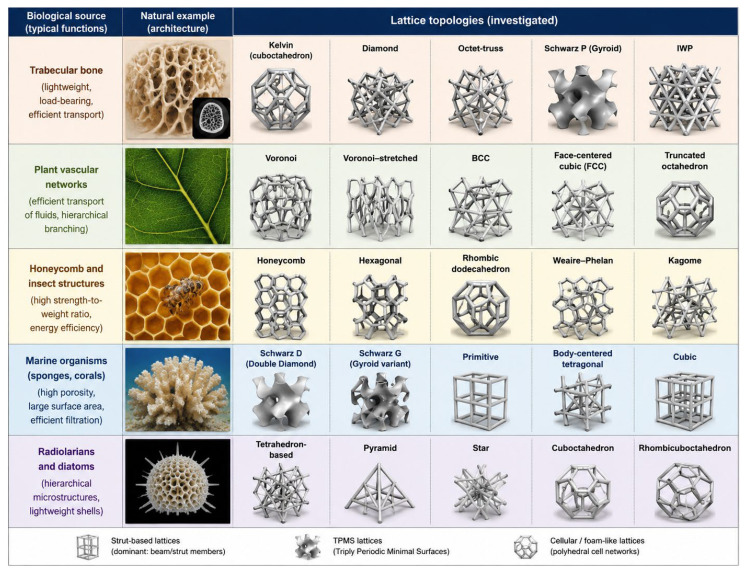
Biomimetic classification of the investigated cellular metamaterials. The analyzed lattice topologies were grouped according to their geometric similarity to naturally occurring cellular architectures, including cancellous bone, plant vascular networks, honeycomb structures, and other biologically inspired porous systems [schematic illustration generated using Stable Diffusion].

These biological principles have inspired the development of architected cellular metamaterials manufactured using additive manufacturing technologies. In contrast to conventional materials, where thermal properties are primarily determined by chemical composition, cellular metamaterials enable the tailoring of effective thermal conductivity through geometric design and topology optimization, as shown in Figure 1 [43].

The present study follows a functional biomimetic design approach. The investigated lattice architectures do not represent direct replicas of specific biological organisms; instead, they reproduce key functional characteristics observed in natural cellular materials, including lightweight porous construction, interconnected transport pathways, efficient material distribution, and topology-controlled transport mechanisms. Consequently, the investigated open-cell cellular metamaterials may be classified as structural and functional biomimetic systems, where biological optimization principles are transferred into engineered thermal-management structures through architected geometry and additive manufacturing (Table 1).

### 1.5. Additive Manufacturing as a Method for Fabricating Bionic Cellular Structures

Traditional manufacturing processes are often unable to accurately reproduce the complex and highly sophisticated nature of biological systems. Recently, three-dimensional (3D) printing has emerged as one of the most powerful technologies for rapid design, prototyping, and manufacturing. Additive manufacturing (AM) represents an efficient approach for fabricating complex biologically inspired metallic and polymeric structures due to its ability to deposit material layer by layer [8,40,52]. 3D printing enables the fabrication of intricate and geometrically complex structures capable of maximizing surface area and enhancing heat-transfer performance. Complex geometries and advanced internal architectures constitute some of the key advantages of additive manufacturing technologies. These capabilities make it possible to produce highly customized and sophisticated designs that are difficult or impossible to achieve using conventional manufacturing methods. The incorporation of biomimetic concepts can be particularly observed in the application of cellular and lattice structures, which generally exhibit not only optimized stiffness-to-weight ratios and lightweight characteristics, but also favorable thermal and acoustic properties.

The analysis of the current state of the art clearly indicates that, despite the rapid development of research on thermal metamaterials and cellular structures, a significant gap still exists between material architecture design, theoretical modeling, and the reliable identification of thermal properties under real operating conditions. In particular, there is still a lack of comprehensive approaches integrating the design of cellular structures as thermal metamaterials fabricated using additive manufacturing technologies (3D printing) with in situ diagnostic methods capable of validating theoretical models and assessing their engineering applicability.

This gap is of considerable importance for environmental engineering and energy-related applications, where materials with controlled thermal transport properties must satisfy stringent operational requirements. Bridging this gap requires the integration of theoretical and experimental methodologies within a coherent research framework, which constitutes the primary objective of the investigations presented in this study.

The state-of-the-art analysis clearly indicates that, despite the intensive development of research on thermal metamaterials and cellular structures, a significant gap still exists between material architecture design, theoretical modeling, and reliable identification of thermal properties under real operating conditions. In particular, coherent approaches that combine the design of cellular structures as additively manufactured thermal metamaterials with in situ diagnostic methods enabling model verification and assessment of engineering applicability remain insufficiently developed.

Recent studies have shown that architected thermal metamaterials may exhibit strongly anisotropic heat-transport properties resulting from the topology of the cellular structure and the spatial arrangement of structural elements. Such anisotropic thermal behavior has been used to realize thermal cloaks, thermal concentrators, heat-flux guiding systems, and structures enabling controlled heat redistribution. Appropriate tailoring of the effective thermal conductivity tensor through geometric design is therefore one of the fundamental mechanisms enabling advanced metamaterial functionality. Despite significant progress in this field, experimental validation of the anisotropic thermal properties of additively manufactured cellular metamaterials remains limited, particularly under conditions representative of real engineering applications.

This gap is particularly important for environmental engineering and energy applications, where materials with controlled heat transport must satisfy rigorous operational requirements. Addressing this gap requires the integration of theoretical and experimental tools within a coherent research framework, which is implemented in the present study.

## 2. Materials and Methods

### 2.1. Mathematical Model of Heat Transfer in Cellular Structures

The cellular metamaterials considered in this study are heterogeneous media in which heat transfer occurs through a coupled system involving conduction through the solid skeleton and, depending on the operating conditions, through the gaseous phase, with possible contributions from internal thermal radiation and convection within the pores or the solid phase. Furthermore, the solid skeleton itself may consist of a combination of different materials, such as polymers and natural (biodegradable) materials, or may incorporate phase-change materials (PCMs) [5,6,8]. Consequently, it is not physically justified to associate thermal conductivity solely with the properties of the bulk solid material. Instead, it becomes necessary to introduce effective thermal properties that depend on the geometry, topology, and characteristic scale of the cellular architecture.

The detailed derivation of the variational formulation, two-scale homogenization procedure, STL-based geometric extraction, permeability formulation, and transient extension is provided in the Supplementary Materials. Only the governing equations and final model components required to interpret the results are presented in the main manuscript, as well as in the Supplementary Materials, Sections S1–S15.

Porosity:

(1) $$\varepsilon =\frac{V_{p}}{V_{cell}}$$

Relative density:

(2) $$\rho_{r}=\frac{V_{s}}{V_{cell}}=1-\varepsilon$$

Steady-state heat conduction equation:

(3) $$\nabla \cdot \left(k\nabla T\right)=0$$

Effective thermal conductivity tensor:

(4) $$K_{eff}=\begin{array}{ccc} k_{xx} & k_{xy} & k_{xz}\\ k_{yx} & k_{yy} & k_{yz}\\ k_{zx} & k_{zy} & k_{zz} \end{array}$$

Fourier’s law for anisotropic media:

(5) $$q_{i}=-K_{ij}\frac{\partial T}{\partial x_{j}}$$

Isotropic case:

(6) $$K_{eff}=k_{\left(eff\right)}I$$

Components of effective thermal conductivity:

(7) $$k_{eff}^{op}=k_{cond,s}+k_{cond,g}+k_{rad}+k_{conv,eff}$$

The effective thermal conductivity of the open-cell metamaterial is assumed to be the sum of four heat-transfer mechanisms: conduction through the solid skeleton, conduction through the gas phase, thermal radiation inside the pore network, and topology-dependent convective transport. This decomposition allows individual contributions to be quantified separately and provides a physically interpretable framework for analyzing architected cellular materials.

Heat conduction through the lattice skeleton:

(8) $$k_{cond,s}=k_{s}[\left(\frac{1}{3}\right)\rho_{r}+\left(\frac{2}{3}\right)\rho_{r}^{\frac{3}{2}}]$$

This relationship originates from cellular-solid theory and accounts for the reduction in conductive pathways caused by increasing porosity. The first term represents direct strut conduction, whereas the second term accounts for node connectivity effects within the cellular network.

Simplified expression for highly porous structures:

(9) $$k_{cond,s}\approx \frac{k_{s}\left(1-\varepsilon \right)}{3}$$

Gas-phase conduction with tortuosity correction:

(10) $$k_{cond,g}=\left(\frac{\varepsilon }{\tau_{g}}\right)k_{g}$$

Gas-phase conduction is reduced by the tortuous geometry of the pore network. The tortuosity factor reflects the increased path length followed by heat flow within the interconnected pore structure.

Hydraulic pore diameter:

(11) $$D_{h}=\frac{4V_{p}}{A_{s}}$$

Hydraulic diameter expressed as a function of porosity and specific surface area:

(12) $$D_{h}=\frac{4\varepsilon }{S_{v}}$$

Rayleigh number based on hydraulic diameter:

(13) $$Ra_{D_{h}}=\frac{g\beta \Delta TD_{h}^{3}}{\nu \alpha_{g}}$$

The hydraulic diameter is adopted as the characteristic length scale because it better represents the actual geometry of irregular interconnected pores than the nominal unit-cell size. Consequently, the Rayleigh number directly reflects topology-dependent buoyancy effects.

Convection suppression criterion for a thermally stable configuration:

(14) $$Ra_{D_{h}}<Ra_{cr}$$

Critical Rayleigh number:

(15) $$Ra_{cr}\approx 1708$$

Effective Nusselt number:

(16) $$Nu_{eff}=1+\eta_{top}\eta_{w}\left(Nu_{D_{h}}-1\right)$$

The coefficients η_top_ and η_w_ account for topology-induced suppression of buoyancy-driven flow and boundary-condition effects. Their values were selected on the basis of permeability characteristics reported for open-cell cellular materials and available experimental observations from porous-media heat transfer studies.

Convective contribution:

(17) $$k_{conv,eff}=\left(\frac{\varepsilon }{\tau_{g}}\right)\left(Nu_{eff}-1\right)k_{g}$$

For the top-to-bottom configuration:

(18) $$Nu_{eff}\approx 1$$

Resulting convective contribution:

(19) $$k_{conv,eff}\approx 0$$

Mean absolute temperature:

(20) $$T_{m}=\frac{T_{hot}+T_{cold}}{2}$$

Effective radiative length:

(21) $$L_{r}=\chi_{r}D_{h}$$

Radiative contribution:

(22) $$k_{rad}=4\varepsilon_{rad}\sigma T_{m}^{3}L_{r}$$

The radiative contribution is obtained from the Rosseland approximation and therefore increases proportionally to the third power of the mean absolute temperature. This mechanism becomes increasingly important at elevated temperatures and for large pore sizes.

Temperature dependence of thermal radiation:

(23) $$\frac{k_{\left(rad\right)\left(T_{m,2}\right)}}{k_{\left(rad\right)\left(T_{m,1}\right)}}={\left(\frac{T_{m,2}}{T_{m,1}}\right)}^{3}$$

Final effective thermal conductivity mode:

(24) $$k_{eff}^{op}=k_{s}\left[\left(\frac{1}{3}\right)\rho_{r}+\left(\frac{2}{3}\right)\rho_{r}^{\frac{3}{2}}\right]+\left(\frac{\varepsilon }{\tau_{g}}\right)k_{g}+4\varepsilon_{rad}\sigma T_{m}^{3}L_{r}+\left(\frac{\varepsilon }{\tau_{g}}\right)\left(Nu_{eff}-1\right)k_{g}$$

Simplified model for the top-to-bottom validation configuration:

(25) $$k_{eff}^{op}\approx k_{cond,s}+k_{cond,g}+k_{rad}$$

Contribution of solid-phase conduction:

(26) $$C_{s}=\left(\frac{k_{cond,s}}{k_{eff}^{op}}\right)\times 100\%$$

Contribution of gas-phase conduction:

(27) $$C_{g}=\left(\frac{k_{cond,g}}{k_{eff}^{op}}\right)\times 100\%$$

Contribution of thermal radiation:

(28) $$C_{rad}=\left(\frac{k_{rad}}{k_{eff}^{op}}\right)\times 100\%$$

Contribution of convection:

(29) $$C_{conv}=\left(\frac{k_{conv,eff}}{k_{eff}^{op}}\right)\times 100\%$$

Sum of contributions:

(30) $$C_{s}+C_{g}+C_{rad}+C_{conv}=100\%$$

Relative model error:

(31) $$Error=\left(\frac{\left|k_{model}-k_{exp}\right|}{k_{exp}}\right)\times 100\%$$

Model–experiment deviation:

(32) $$\Delta k=k_{model}-k_{exp}$$

**Figure 2:**
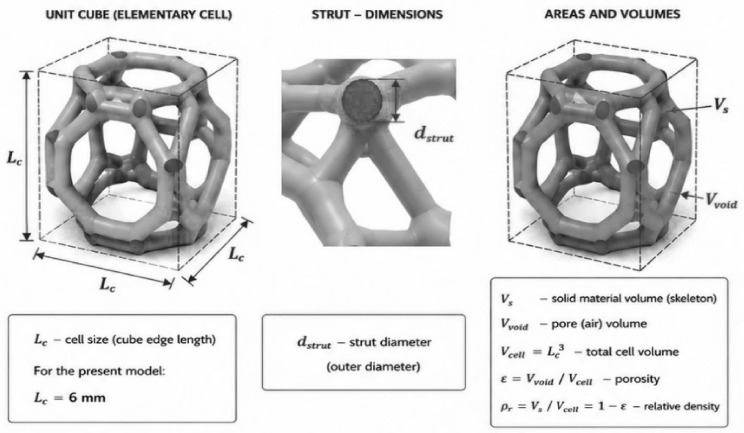
Geometrical parameters of the Kelvin-cell-based cellular structure.

For each lattice structure obtained from an STL file, the model should account not only for porosity and relative density, but also for specific surface area, hydraulic diameter, pore inlet area, permeability, and gas-phase tortuosity. Calculations for a representative Kelvin cell are presented below in Figure 2.

**Figure 3:**
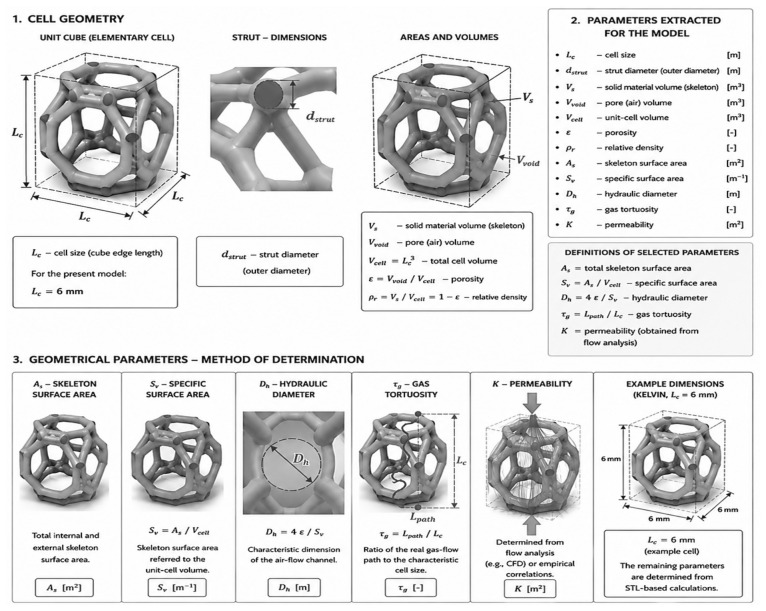
Geometrical and transport parameters extracted from the STL geometry for a representative Kelvin cell.

The parameters that must be extracted from each STL geometry are summarized in Figure 3.

### 2.2. Biomimetic Interpretation of the Proposed Mathematical Model

Although the governing equations employed in the present study are based on classical heat-transfer theory, the proposed mathematical framework incorporates several geometric descriptors that are commonly used to characterize biological porous systems [53,54]. Parameters such as porosity, relative density, specific surface area, hydraulic diameter, tortuosity, and permeability describe the transport architecture of both natural cellular materials and engineered lattice metamaterials [54,55,56].

From a biomimetic perspective, the proposed model does not attempt to reproduce specific biological geometries directly [41,42]. Instead, it captures fundamental biological design principles associated with hierarchical porous networks and topology-controlled transport pathways. Consequently, the effective thermal conductivity predicted by the model emerges from architectural characteristics analogous to those observed in cancellous bone, wood, coral skeletons, and other naturally occurring cellular materials [36,41,48]. This approach follows the concept of functional biomimicry, where biological transport mechanisms are reproduced through engineered geometry rather than through direct replication of biological forms.

### 2.3. Validation of a Mathematical Model Using Experiments

In the author’s previous work [57], prototype thermal-insulation materials were designed as periodic open-cell lattice structures based on the Kelvin cell and intended for thermal-insulation applications. The analyzed structures were modeled as sandwich composites with a porous cellular core. The investigated specimens differed in pore diameter L_c_, porosity ε, and the number of layers n. Four pore diameters were considered: d = 4 mm, 6 mm, 8 mm, and 10 mm. For each pore diameter, two porosity levels were analyzed: ε = 0.95 and ε = 0.90. The specimens were manufactured using selective laser sintering (SLS). The printing process was carried out using a Sinterit Lisa SLS 3D printer (Sinterit Sp. z o.o., Kraków, Poland). Thermal-property measurements were performed using a thermoelectric version of the Poensgen apparatus (sometimes named hot and cold plate) apparatus, which was built at the Wrocław University of Science and Technology. The specimen dimensions were 50 mm × 50 mm × 20 mm. The investigations were conducted for two heat-flow configurations. Variant I—top-to-bottom heat transfer. In the first configuration, heat flowed from the upper surface toward the lower surface. The upper surface of the specimen was heated, whereas the lower surface was cooled: $T_{top}$ = +20 °C and $T_{bottom}$ = −20 °C. This configuration corresponded to a gravitationally stable thermal system. Variant II—bottom-to-top heat transfer. In the second configuration, heat flowed from the lower surface toward the upper surface. The lower surface was heated, while the upper surface was cooled: $T_{bottom}$ = +20 °C and $T_{top}$ = −20 °C. Such a configuration enabled the development of natural convection inside the open-cell structures. The applied temperature difference was: ∆T = 40 K which corresponds to typical operating conditions of thermal-insulation systems used in building engineering, refrigeration, and frozen-food transportation. Before recording the measurements, thermal equilibrium conditions had to be achieved. The stabilization time ranged from approximately 30 to 50 min after placing the specimen inside the testing apparatus.

The results obtained using the proposed mathematical model and the experimental investigations are presented in Table 2 for the top-to-bottom heat-flow configuration and in Table 3 for the bottom-to-top heat-flow configuration. For the experimentally validated top-to-bottom configuration, buoyancy-driven convection is strongly suppressed and therefore Nu_eff_ ≈ 1.

The analysis of both the mathematical model and the experimental results indicates that, for open-cell structures, there exists a characteristic geometric threshold beyond which convective heat transfer begins to exert a noticeable influence on the overall thermal transport behavior. The region near L_c_ = 6 can therefore be interpreted as a transition zone between conduction-dominated and mixed conduction–convection transport regimes. Although the nominal cell size L_c_ was initially used as a characteristic geometric parameter, the hydraulic diameter D_h_ provides a more physically meaningful length scale for describing buoyancy-driven flow in open-cell lattices. The effective Rayleigh number is therefore more appropriately interpreted as being governed by D_h_, permeability, and pore connectivity rather than by the nominal cell size alone. As Lc increases, the corresponding hydraulic diameter and permeability increase, resulting in higher effective Rayleigh numbers and the gradual emergence of local convective transport. For small cellular architectures, particularly around L_c_ = 4 mm, the structures exhibit narrow flow channels, high flow resistance, increased tortuosity, small hydraulic diameters, and consequently low effective Rayleigh numbers.

Under such conditions, natural convection remains practically suppressed: Nu ≈ 0 and heat transfer is governed predominantly by conduction and thermal radiation. As the characteristic cell size approaches $L_{c}$ = 6 mm, larger pore channels begin to form, resulting in lower hydraulic resistance, increased permeability, and increased hydraulic diameter, and consequently increasing effective Rayleigh numbers. This regime may therefore be interpreted as a geometric transition region. For structures with $L_{c}$ > 6 mm, the buoyancy effects become significantly more pronounced because Ra ∝ $D_{h}^{3}$, which means that even a relatively small increase in the hydraulic diameter may produce a substantial increase in buoyancy-driven flow intensity. In the classical Rayleigh–Bénard problem, the critical Rayleigh number is given by $Ra_{crit}$ ≈ 1708. However, Kelvin-type open-cell lattices cannot be treated as simple planar channels or homogeneous fluid layers. Instead, they form highly interconnected three-dimensional networks of air-filled cells. Consequently, local vortices, microscale circulation loops, and localized recirculation zones may appear significantly earlier than predicted by the classical Rayleigh–Bénard instability criterion. Therefore, the transition between purely conductive and convection-assisted transport should not be interpreted as a single sharp threshold, but rather as a gradual transition associated with the evolving topology and hydraulic connectivity of the cellular network.

According to the experimental results reported in Ref. [57], convection remains negligible for structures with $L_{c}$ <= 4–5 mm. Around $L_{c}$ = 6 mm, partial convection effects begin to emerge, including localized gas motion and an increasing contribution of the convective component $k_{conv}$. For structures with $L_{c}$ >= 8–10 mm, convective effects may significantly influence the effective thermal conductivity, particularly in highly porous structures and under thermally unstable configurations. Even though the Rayleigh number increases for larger cellular architectures, the top-to-bottom heat-flow configuration remains gravitationally stable. Consequently, fully developed Rayleigh–Bénard convection does not occur. Nevertheless, local microscale recirculation phenomena, small vortices, lateral pore-scale flow, and mixed transport mechanisms may still develop inside the interconnected pore network. This behavior corresponds precisely to a transitional transport regime.

In summary, both the experimental and analytical results indicate the existence of a transition region for open-cell lattice structures near $L_{c}$ = 6 mm. Below this characteristic cell size, heat transfer is dominated primarily by conduction through the solid skeleton, gas-phase conduction, and thermal radiation, whereas convective effects remain negligible due to the limited hydraulic diameter of the pores and the high flow resistance.

As the characteristic cell size increases beyond approximately 6 mm, the effective hydraulic diameter and permeability of the structure increase significantly, leading to a rapid increase in the effective Rayleigh number. Consequently, weak local convection and recirculatory gas motion begin to contribute noticeably to the effective thermal conductivity. The STL-based model demonstrated that gas-phase conduction constitutes a dominant contribution in highly porous structures such as Kelvin lattices. Combined with thermal radiation and a partially suppressed convective component, the proposed model accurately predicts the effective thermal conductivity observed in the experimental investigations.

These findings confirm that neglecting gas-phase conduction leads to a significant underestimation of heat-transfer capability in open-cell cellular metamaterials.

The satisfactory agreement obtained between the analytical predictions and the experimental measurements motivated the author to extend the proposed mathematical framework to other lattice topologies.

### 2.4. Materials and Design of Cellular Thermal Metamaterials for Additive Manufacturing

In investigations of architected materials, a common source of apparent inconsistencies arises from comparing results obtained for different specimen orientations and different thermal-loading configurations. From a formal perspective, this phenomenon is a direct consequence of the transformation of the thermal-conductivity tensor under a change in the reference coordinate system [6]. This implies that a measurement performed in a single direction does not constitute a complete characterization of the material if the material exhibits anisotropic behavior. In materials possessing significant off-diagonal components of the conductivity tensor, situations may occur in which the heat flux vector is not collinear with the temperature gradient.

Consequently, in the interpretation of in situ thermal diagnostics, it becomes necessary to distinguish between the “material effect” and the “boundary-condition and contact effect” [6]. These considerations are of direct relevance for measurement techniques based on guarded hot-plate and plate-apparatus systems, as well as for procedures implemented in thermal-testing standards, where isotropic material behavior is often implicitly assumed [6,58,59,60,61,62].

In the evaluation of thermal properties, standardized measurement methods play a crucial role, particularly with respect to the comparability of results and their transferability to practical engineering applications. In laboratory investigations, guarded hot-plate apparatuses and steady-state methods compliant with ISO 8301 [62] are widely employed. For in situ applications, the reference standard is provided by methods for measuring the thermal resistance and thermal transmittance of building components in accordance with ISO 9869-1 [61]. At the same time, it should be emphasized that cellular metamaterials frequently do not fully satisfy the assumptions underlying classical measurement methods. Such materials may exhibit anisotropic thermal behavior, temperature distributions strongly dependent on local geometry, and pronounced sensitivity to thermal contact conditions and surface roughness [6]. Consequently, the interpretation of the experimental results should be directly linked to an appropriate physical model and, where justified, supported by numerical simulations.

Within the framework of transformation-based approaches and the broader field of thermal metamaterials, considerable attention has been devoted to the realization of functionalities such as thermal cloaks, thermal concentrators, and thermal rotators. From an engineering perspective, however, the critical challenge lies in transitioning from proof-of-concept demonstrations to the parametric design of architectures in a practically useful manner, namely by establishing how geometric modifications influence $k_{eff}$ and how this relationship can subsequently be inverted through inverse-design methodologies [9,63,64,65,66]. In this context, cellular structures—including TPMS architectures, Kelvin cells, layered systems, and bioinspired geometries—become highly effective platforms for the realization of “engineering thermal metamaterials.” Their significance arises from the fact that they can be fabricated using additive manufacturing technologies (AM/3D printing) [40,67,68,69,70,71,72,73], parametrically controlled, experimentally validated, and subsequently implemented into engineering-scale models and technical systems.

#### 2.4.1. Methodology for Designing Bionic Cellular Structures in the Rhino/Grasshopper Environment

The parametric design of strut-based lattice structures was based on the fundamental separation of the description into two distinct layers: a geometric layer, defined as a set of nodes V represented by points in space $v_{i}$ = ($x_{i},\;y_{i},\;z_{i}$), and a topological layer, defined as a set of struts E represented by pairs of indices (i,j), indicating which nodes are connected.

In the Grasshopper dla Rhino 7 environment, this approach is implemented by generating lists or tree structures of points (geometry), followed by the creation of neighborhood relationships through predefined offsets within the data structure, which determine the pairing of points A–B and consequently the generation of line segments (struts) [65,74,75,76,77,78,79,80,81,82].

Such an approach minimizes the need for manual generation of struts “cell by cell” and enables efficient scaling of the model to a large number of repeating cellular units. As a result, controlled anisotropy of the effective thermal-conductivity tensor $k_{eff}$ can be achieved, enabling the design of conductivity gradients and structures exhibiting spatially varying thermal functions within a single component.

Lattice-structure design inherently requires compromises between several competing factors, including topological complexity (number of strut classes and node coordination degrees), functional control (thermal isotropy/anisotropy and thermal stability), and manufacturing feasibility (3D printing constraints, tolerances, and repeatability).

**Figure 4:**
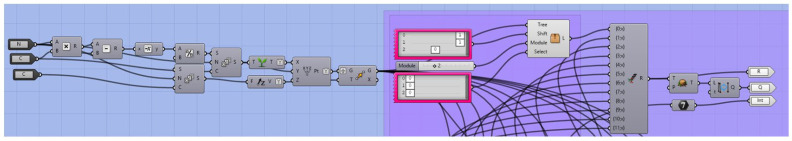
Example of a parametric algorithm for the generation of an all face-centered cubic lattice structure.

**Figure 5:**
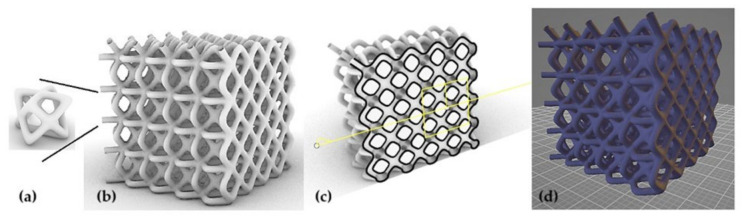
Cellular material with an open-cell core structure based on the all face-centered cubic unit cell: (**a**) single unit cell, (**b**) assembly of interconnected cells, (**c**) cross-sectional view of the cellular assembly, (**d**) view of the cellular assembly in the generated *.stl* file.

The formal graph-based description enables these design decisions to be performed systematically rather than intuitively, constituting one of the key elements of the proposed methodology. An example of the computational workflow used for generating lattice structures is presented in Figure 4 and Figure 5.

**Figure 6:**
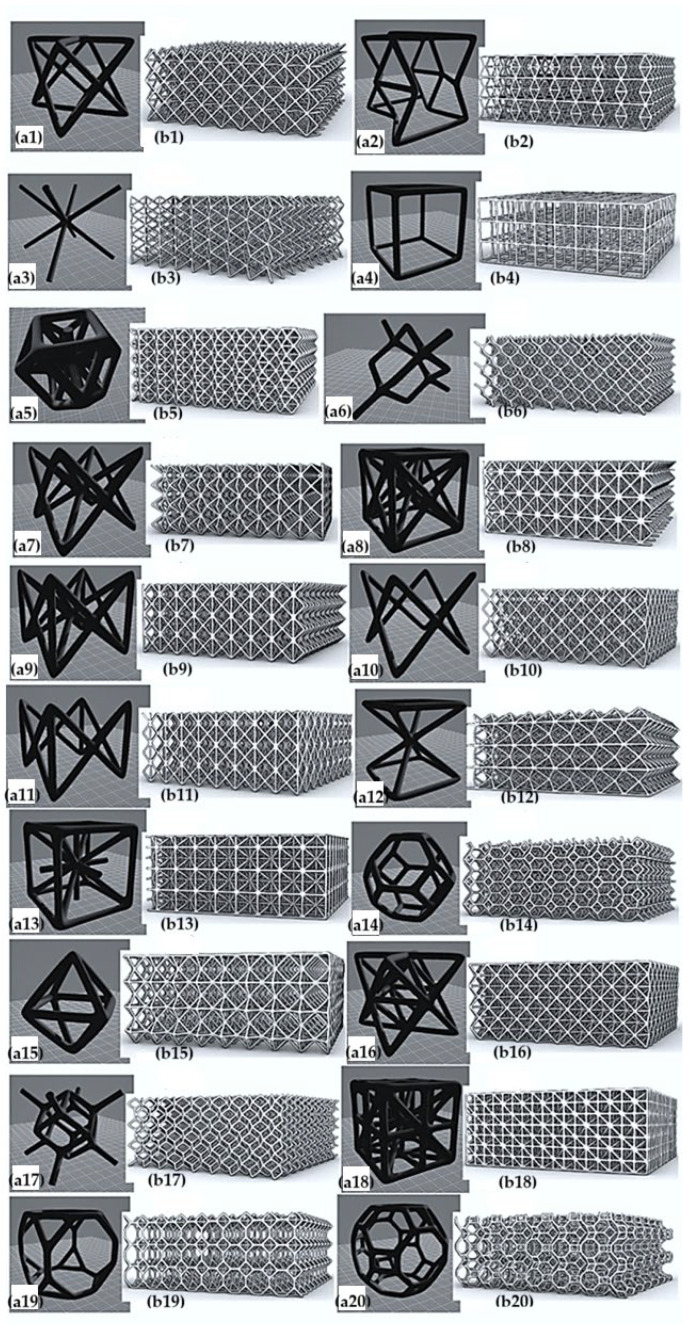
Types of skeletal lattice structures used in the investigations for epsilon = 95% and $L_{c}$ = 6 mm, (**a1**–**a20**) single unit cell; (**b1**–**b20**) structural panel; (**a1**,**b1**) all face-centered cubic, (**a2**,**b2**) auxetic, (**a3**,**b3**) body-centered cubic (BCC), (**a4**,**b4**) cube, (**a5**,**b5**) cuboctahedron Z, (**a6**,**b6**) diamond, (**a7**,**b7**) face-centered cubic (FCC), (**a8**,**b8**) FBCCXYZ, (**a9**,**b9**) FCCZ, (**a10**,**b10**) FBCC, (**a11**,**b11**) FBCCZ, (**a12**,**b12**) G7, (**a13**,**b13**) iso truss, (**a14**,**b14**) Kelvin, (**a15**,**b15**) octahedron, (**a16**,**b16**) octet truss, (**a17**,**b17**) rhombic dodecahedron, (**a18**,**b18**) tetrahedron-based, (**a19**,**b19**) truncated cube, (**a20**,**b20**) truncated cuboctahedron.

For both the theoretical analysis and the experimental investigations, additively manufactured cellular structural panels were considered. The analyzed structures were composed of a UV white resin as the base material and air filling the cellular pore space. The cellular skeleton consisted of an open-cell foam-like core architecture. The total thickness of the structural panel was H = 20 mm. The characteristic cell diameter of the structural panel was $L_{c}$ = 6 mm (considered in the experiment) and $L_{c}$ = 4–10 mm (considered in the mathematical model). The strut diameter of the cellular framework was d = 0.4 mm. The porosity of the structural panel was in the range of ε = 50–95%, including the following porosity levels: 50%, 70%, 90%, and 95% (considered in the mathematical model), and ε = 95% (considered in the experiment). The heat flow was oriented horizontally from the top toward the bottom surface of the specimen. The open-cell core architecture was based on various types of skeletal lattice structures, as presented in Figure 6.

Thermal metamaterials encompass a broad class of materials and structures in which the direction, intensity, and localization of heat flux can be deliberately controlled [10,11]. Several major research directions in this field can be identified in the literature.

The first direction concerns so-called transformation-based materials, in which the spatial distribution of thermal conductivity is designed using coordinate transformations that enable functionalities such as thermal cloaks, heat concentrators, and thermal rotators [3]. Although this approach is mathematically elegant, it often leads to solutions that are difficult to realize technologically due to the requirement for strongly anisotropic and spatially heterogeneous material properties.

The second research direction involves metamaterials based on periodic structures, in which heat-transfer control is achieved through the appropriate design of elementary-cell geometry. In this approach, thermal conductivity is treated as an effective property resulting from the homogenization of local transport phenomena occurring within materials possessing complex microarchitectures [5]. Such an approach is particularly attractive from an engineering perspective because it enables the use of conventional base materials, while the metamaterial functionality is achieved exclusively through structural design.

The third research direction focuses on dynamic phenomena and non-Fourier heat-transfer models, in which wave-like effects may occur, including so-called thermal waves and forbidden energy-transport band gaps [5,10,29]. Although these phenomena are highly relevant at micro- and nanoscale dimensions and over very short time scales, their practical implementation in engineering applications remains the subject of ongoing discussion and intensive research.

**Figure 7:**
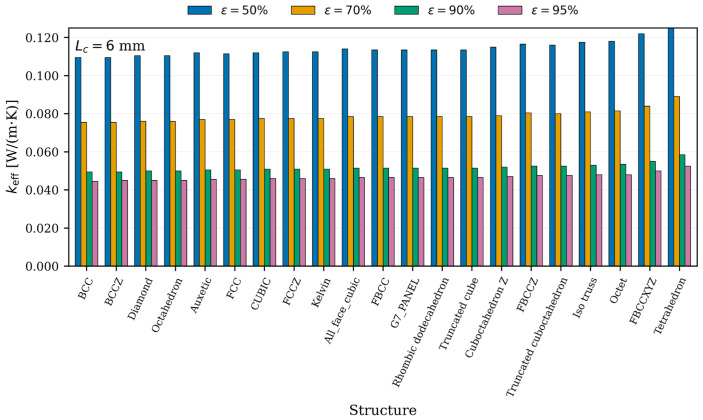
Influence of porosity on the effective thermal conductivity of the investigated lattice metamaterials. The results represent average values obtained for porosities of 50%, 70%, 90%, and 95% for a unit-cell size of Lc = 6 mm.

**Figure 8:**
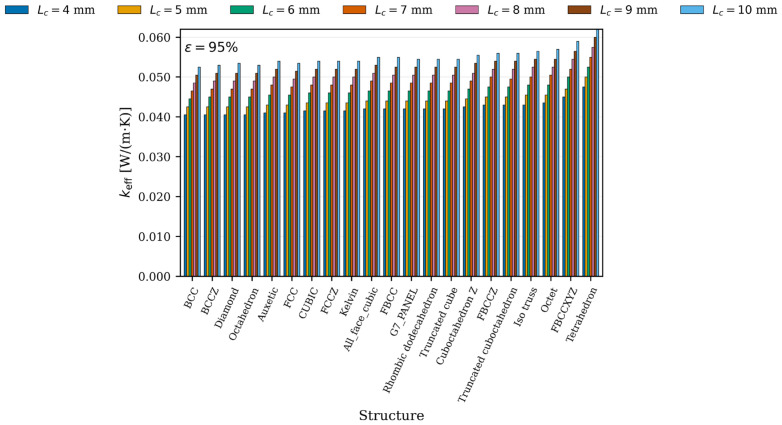
Influence of unit-cell size on the effective thermal conductivity of the investigated lattice metamaterials. The results are shown for unit-cell sizes ranging from 4 mm to 10 mm and for porosities of 95%.

**Figure 9:**
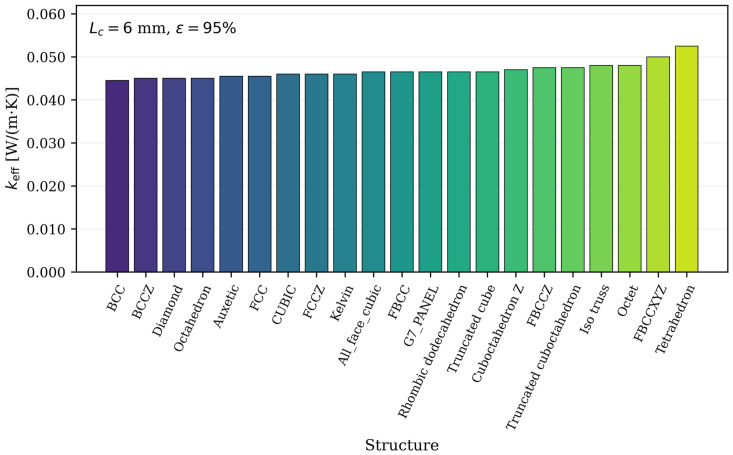
Ranking of investigated lattice topologies according to the effective thermal conductivity k_eff_ determined for a unit-cell size of Lc = 6 mm and a porosity of 95%. Structures are arranged from the lowest to the highest thermal conductivity.

The calculations were performed using the extended model for open-cell skeletal lattice structures with characteristic cell sizes $L_{c}$ = 4, 5, 6, 7, 8, 9, 10 mm and porosity levels of 50%, 70%, 90%, and 95% for two different heat-flow configurations. The obtained computational results are presented in the tables. A complete calculation matrix was generated individually for each structure: 20 structures × 7 cell diameters × 4 porosity levels, resulting in a total of 560 computational variants. The extended model for open-cell structures presented in Section 2.1 was adopted throughout the analysis. The computational results are presented in Table S1 (in the Supplementary Materials). The most important results are also presented in Figure 7, Figure 8 and Figure 9.

Figure 7 compares the effective thermal conductivity of all investigated structures for porosity levels of 50%, 70%, 90%, and 95%. A systematic reduction in thermal conductivity can be observed with increasing porosity for all investigated topologies. The reduction is primarily associated with the decreasing volume fraction of the solid phase and the increasing contribution of the gas-filled pore space. However, the rate of reduction differs significantly among topologies. Some structures exhibit a relatively gradual decrease in k_eff_, whereas others show a much stronger sensitivity to porosity changes. This behavior demonstrates that porosity and topology are strongly coupled parameters that jointly determine the thermal performance of lattice metamaterials. At high porosity levels (ε = 90–95%), the differences between individual topologies become particularly pronounced. Under these conditions, gas-phase conduction and radiative heat transfer contribute significantly to the total heat flux, while the geometry-dependent connectivity of the remaining solid skeleton becomes increasingly important.

Figure 8 illustrates the influence of unit-cell size on effective thermal conductivity for all investigated lattice structures. For most topologies, an increase in cell size from 4 mm to 10 mm leads to a gradual increase in effective thermal conductivity. This trend is associated with the enlargement of pore dimensions and hydraulic diameter, which modifies both radiative transport and fluid-flow characteristics within the cellular network. For small unit cells, heat transfer is dominated by solid conduction and gas conduction. As the cell size increases, the characteristic radiative path length also increases, resulting in a greater radiative contribution to the overall thermal conductivity. In open-cell structures, larger pores additionally increase permeability and may promote local buoyancy-driven gas motion. A particularly important observation is that the influence of cell size varies considerably between different topologies. Structures possessing highly interconnected transport pathways exhibit stronger sensitivity to geometric scaling than architectures characterized by more isotropic or tortuous networks. Consequently, unit-cell size should be regarded as an independent design parameter alongside porosity and topology during the development of thermal metamaterials.

Figure 9 presents the ranking of all investigated lattice metamaterials according to their effective thermal conductivity determined for a unit-cell size of Lc = 6 mm and a porosity of 95%. The results clearly demonstrate that lattice topology exerts a significant influence on thermal transport even when the overall porosity remains constant. The investigated structures exhibit noticeable differences in effective thermal conductivity despite possessing identical constituent material properties and comparable relative densities. The lowest values of k_eff_ were observed for topologies characterized by highly tortuous heat-transfer pathways and reduced solid-phase connectivity. In contrast, structures containing more direct conductive pathways between opposite faces of the unit cell exhibited increased thermal conductivity.

The calculations performed using the proposed mathematical model for open-cell structures demonstrated that the effective thermal conductivity strongly depends on porosity. For each analyzed cell size, increasing the porosity from 50% to 95% resulted in a monotonic decrease in k_eff_ primarily due to the reduced contribution of the solid phase and the gas phase to the overall heat-transfer process. For a porosity level of 50%, the predicted thermal-conductivity values were within the range of approximately k_eff_ = 0.125–0.133 W/(m·K), whereas for a porosity of 95%, the values decreased to approximately k_eff_ = 0.049–0.057 W/(m·K). These results indicate that porosity constitutes one of the dominant parameters governing heat transport in cellular metamaterials, while the influence of topology is reflected in the dispersion of thermal-conductivity values observed within each porosity level.

This observation confirms that porosity alone cannot be considered a sufficient descriptor of thermal insulation performance. Instead, the spatial arrangement of the cellular network, strut orientation, node connectivity, and topology-dependent transport pathways play a dominant role in determining the overall thermal response of architected cellular metamaterials. From a biomimetic perspective, these results are consistent with natural porous systems, where transport properties are controlled not only by void fraction but also by the architecture of the internal network. Similar transport optimization mechanisms can be found in cancellous bone, wood vascular systems, coral skeletons, and other naturally occurring cellular materials.

#### 2.4.2. In Situ Experimental Setup for Thermal Conductivity

**Figure 10:**
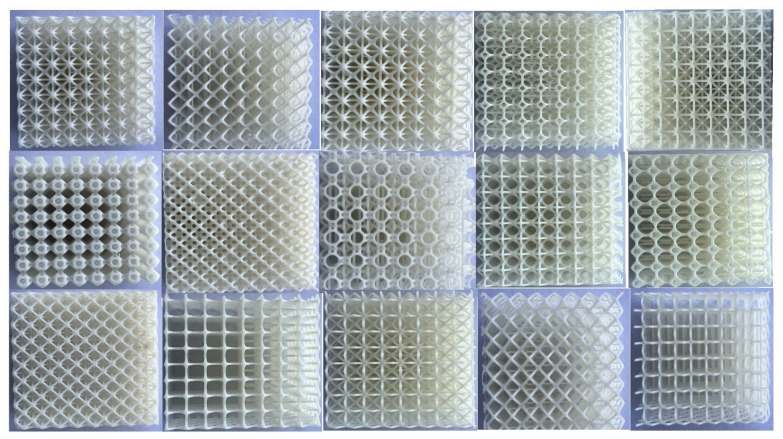
Representative printed metamaterial specimens with different open-cell internal core architectures.

In order to validate the proposed mathematical model, experimental investigations were conducted on specimens fabricated using a Jupiter mSLA 3D printer (Shenzhen Elegoo Technology Co., Ltd., Shenzhen, Guangdong, China. The experiments focused on determining the effective thermal conductivity coefficient k. The selected specimens were characterized by a cell size of $L_{c}$ = 6 mm and a porosity of ε = 95%, for which the calculated thermal-conductivity values exhibited the most favorable performance for both analyzed heat-flow configurations. Representative fabricated specimens are presented in Figure 10. The specimen dimensions were 50 mm × 50 mm × 20 mm. The experimental investigations were carried out for a single heat-flow configuration corresponding to top-to-bottom heat transfer. The upper surface of the specimen was heated, whereas the lower surface was cooled $T_{top}$ = +20 °C and $T_{bottom}$ = −20 °C. This configuration corresponded to a gravitationally stable thermal system.

The thermal performance of the fabricated sample was evaluated using a custom-built steady-state experimental apparatus developed at the Department of Energy Conversion Engineering, Faculty of Mechanical and Power Engineering, Wroclaw University of Science and Technology [68,70,73]. The measurement methodology was based on the principles specified in ISO 9869-1:2014 [61] and was adapted for laboratory investigations of highly porous cellular metamaterials. A detailed description of the experimental apparatus, testing methodology, and thermal property evaluation procedure can be found in the author’s previous publications [68,70,73]. In this work, all samples described above were experimentally characterized under identical test conditions.

## 3. Results and Discussion

**Figure 11:**
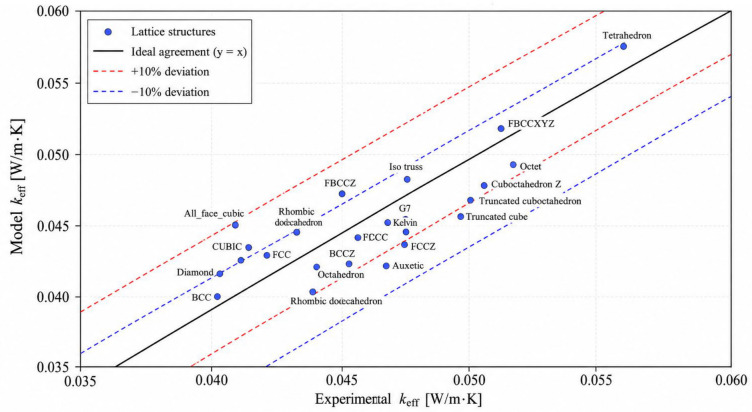
Comparison between experimentally measured and model-predicted effective thermal conductivity values for the investigated lattice metamaterials. The solid line represents perfect agreement (k_model_ = k_exp_), while the dashed lines indicate the ±10% deviation interval.

**Figure 12:**
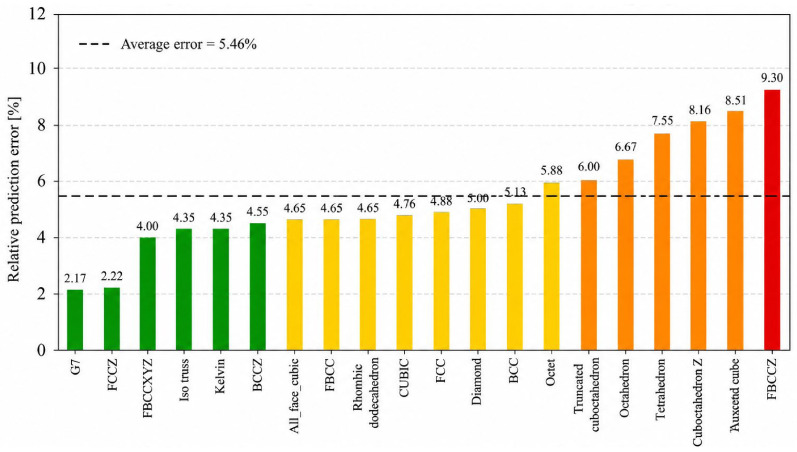
Relative prediction error of the proposed thermal conductivity model for individual lattice topologies. The average deviation between theoretical predictions and experimental measurements equals approximately 5.5%, confirming the validity of the proposed homogenization framework.

The results obtained from the experimental investigations and the mathematical model are presented in Table 4 and Figure 11 and Figure 12.

The experimental investigations demonstrated that the effective thermal conductivity of architected cellular metamaterials depends strongly not only on porosity, but also on the microarchitectural topology of the cellular network.

Figure 11 compares the experimentally measured and theoretically predicted effective thermal conductivity values for all investigated lattice topologies. A strong correlation between the model and the experimental results can be observed. All data points are located within the ±10% deviation band, demonstrating the robustness of the proposed homogenization-based model. The average prediction error equals approximately 5.5%, which is comparable to or lower than values reported in previous studies on architected cellular materials. The agreement confirms that the model adequately captures the combined effects of solid conduction, gas conduction, thermal radiation, and topology-dependent convection.

The relative prediction errors for individual structures are presented in Figure 12. The lowest discrepancies were observed for the G7 and FCCZ topologies, with errors of 2.17% and 2.22%, respectively. The largest deviation was obtained for the FBCCZ structure (9.30%), followed by the auxetic and truncated cube topologies. These discrepancies are primarily attributed to manufacturing-induced geometric deviations, local strut-thickness variations, and STL-parameter uncertainties. Nevertheless, all errors remain below 10%, which is generally considered acceptable for highly porous additively manufactured lattice materials. This level of agreement validates the applicability of the proposed model for topology-driven thermal metamaterial design.

The comparison between the mathematical model and the experimental measurements demonstrated very good agreement for all investigated open-cell lattice topologies manufactured from UV resin. The calculations were performed for a representative cell size of Lc = 6 mm and a porosity of ε = 95%, corresponding to highly porous architected cellular metamaterials. The samples were arranged horizontally, while the heat transfer direction was perpendicular to the specimen plane, i.e., from the upper surface toward the lower surface. Under these conditions, the thermal configuration remained gravitationally stable because the warmer region was located above the colder region. In the top-to-bottom heat-flow configuration, the system is gravitationally stable and classical Rayleigh–Bénard convection cannot fully develop. Consequently, heat transfer is dominated by conduction through the solid skeleton, conduction through the gas phase, and thermal radiation. The effective convective term introduced in the present model represents only local topology-induced gas recirculation and micro-scale flow effects occurring within the interconnected pore network. Therefore, its contribution remains significantly lower than the conductive and radiative components under the investigated experimental conditions. The effective thermal conductivity was therefore described as the sum of conduction through the solid skeleton, gas-phase conduction inside the pores, internal thermal radiation and a calibration correction term accounting for manufacturing imperfections and contact-related effects. The comparison between the model predictions and experimental measurements revealed relative errors ranging from approximately 2% to 9%, with an average deviation of approximately 5.6%. Such agreement is considered highly satisfactory for architected porous materials manufactured by additive technologies, particularly in view of the geometric complexity of the investigated lattices and the unavoidable variability associated with the printing process.

The smallest discrepancies between the model and the experimental results were observed for the G7 and FCCZ structures, where the relative error remained close to 2%. Similarly low deviations were obtained for Kelvin, iso truss and FBCCXYZ lattices. These structures are characterized by relatively regular pore morphology and more uniform heat-flow pathways, which improves the accuracy of homogenization-based descriptions. In such cases, the geometrical descriptors extracted from the STL models, including the relative density, specific surface area and effective pore diameter, were sufficient to reproduce the experimentally observed thermal behavior with high accuracy. Larger deviations were observed for FBCCZ, auxetic, truncated cube and cuboctahedron Z structures, where the relative error approached 8–9%. These discrepancies are most likely associated with local anisotropy of the heat-flow pathways, geometrically complex node regions and non-uniform radiative exchange between internal surfaces. In these structures, the actual radiative view factor and the effective tortuosity of the gas phase may differ from the simplified assumptions adopted in the analytical formulation. Furthermore, additively manufactured polymeric lattices exhibit local micro-porosity, roughness and ligament thickening, especially near node intersections, which may increase the effective thermal conductivity compared with the idealized STL representation.

One of the most important observations is that all investigated structures possessed identical porosity ε = 95%, yet the predicted and experimentally validated effective thermal conductivities varied significantly: 0.041 <= k_eff_ <= 0.057 W/(m·K). This demonstrates that porosity alone is insufficient to characterize the thermal behavior of open-cell metamaterials. Instead, the topology of the cellular architecture, the connectivity of the pore network, the tortuosity of the gas pathways and the internal radiative exchange play equally important roles in determining the overall thermal performance. The lowest effective thermal conductivity values were obtained for BCC, BCCZ, diamond and octahedron lattices. These structures are characterized by smaller effective hydraulic diameters and more tortuous heat-transfer paths, which reduce both gas-phase transport and radiative exchange. In contrast, tetrahedron, FBCCXYZ and octet structures exhibited noticeably higher thermal conductivity values. Their larger pore connectivity and more open transport pathways promote gas conduction and internal radiative transfer, thereby increasing the overall heat transport capability.

The obtained results confirm that the proposed model successfully captures the dominant heat-transfer mechanisms in highly porous open-cell metamaterials. Most importantly, this study demonstrates that effective thermal conductivity cannot be treated solely as a function of porosity, k_eff_ = f(ε), but rather as a complex function of geometry, topology and transport pathways: k_eff_ = f(ρ_r_, S_v_, D_h_, τ_g_, topology, radiation).

The presented approach therefore provides a physically consistent and experimentally validated framework for predicting heat transfer in architected thermal metamaterials produced using additive manufacturing.

### 3.1. Discussion on Thermal Conductivity Anisotropy

The mathematical model developed in the present work assumes isotropic effective thermal conductivity and therefore represents thermal transport using a scalar effective conductivity $k_{eff}$. Such an assumption is commonly adopted in first-order homogenization models [6,83,84] and is particularly appropriate when heat transfer is investigated only along a single principal direction.

However, the lattice structures investigated in this study exhibit different levels of geometric symmetry and therefore may display different degrees of thermal anisotropy [6,34,35]. Highly symmetric structures, such as Kelvin cells, octet-truss lattices, rhombic dodecahedra, and TPMS-derived architectures, are expected to exhibit nearly isotropic thermal transport because conductive pathways are distributed relatively uniformly in three-dimensional space [34,85].

In contrast, body-centered cubic (BCC), face-centered cubic (FCC), tetrahedron-based, and certain anisotropic strut-based architectures contain preferential conductive pathways that may lead to directional differences in thermal conductivity [66,86]. For such structures, the effective thermal conductivity should generally be represented by the tensor. The experimental validation performed in the present study was limited to one principal heat-flow direction corresponding to the vertical top-to-bottom configuration. Consequently, the reported thermal conductivity values should be interpreted as directional effective conductivities rather than complete tensor properties.

A practical experimental verification strategy would involve rotating the specimens and measuring thermal conductivity along three orthogonal directions. The resulting conductivity tensor could then be obtained through numerical homogenization and experimental calibration. The degree of anisotropy could be quantified using the anisotropy ratio. Such investigations would enable the design of anisotropic thermal metamaterials capable of directional heat guiding, thermal concentration, and programmable heat-flow management [86].

Future research should focus on the multidirectional thermal characterization of cellular metamaterials. Such investigations should include thermal conductivity measurements performed along multiple principal directions, determination of the complete effective thermal conductivity tensor, including its off-diagonal components, and validation of anisotropic heat-transfer models through a combination of experimental characterization and numerical homogenization techniques. Furthermore, the integration of additive manufacturing, in situ thermal diagnostics, high-resolution imaging, and inverse-design methodologies has the potential to provide a powerful framework for the development of thermal metamaterials with deliberately tailored anisotropic heat-transfer properties.

### 3.2. Influence of Temperature on Heat-Transfer Mechanisms

The experimental investigations performed in the present study were conducted under thermal conditions corresponding to an average specimen temperature close to 0 °C. Consequently, the experimental validation of the model is limited to a relatively narrow temperature interval representative of thermal-insulation applications.

The conductive contribution of the solid phase is governed by the thermal conductivity of the base material, which may vary with temperature depending on polymer composition and manufacturing conditions. Similarly, the thermal conductivity of the gas phase exhibits temperature dependence, although the variation remains relatively small within the investigated temperature range.

In contrast, the radiative contribution exhibits a significantly stronger temperature dependence. According to the linearized radiation model [83,87]:

(33) $$k_{rad}=4\varepsilon_{rad}\sigma T_{m}^{3}L_{r}$$

the radiative component increases proportionally to the third power of the mean absolute temperature.

For two different temperatures, $T_{m,1}$ and $T_{m,2}$, assuming constant emissivity and radiative path length:

(34) $$\frac{k_{\left(rad\right)\left(T_{m,2}\right)}}{k_{\left(rad\right)\left(T_{m,1}\right)}}={\left(\frac{T_{m,2}}{T_{m,1}}\right)}^{3}$$

This relationship indicates that thermal radiation becomes increasingly important at elevated temperatures. For example, increasing the mean temperature from 273 K to 373 K increases the radiative contribution by approximately:

(35) $${\left(\frac{373}{273}\right)}^{3}\approx 2.55$$

Thus, the radiative component may become more than twice as large as that observed under the experimental conditions investigated in the present study.

Consequently, although the proposed model remains physically valid over a wider temperature range, additional validation is required for high-temperature applications where radiative heat transfer becomes increasingly dominant.

### 3.3. Effects of Additive Manufacturing Defects

Additive manufacturing processes inevitably introduce geometric imperfections that may affect the thermal properties of cellular metamaterials. The most common defects observed in additively manufactured lattice structures include non-uniform strut thickness, surface roughness, strut waviness, deviations from the nominal cross-sectional geometry, and local porosity or defects near nodal regions [88]. Similar manufacturing-related imperfections have also been identified as important factors influencing the accuracy of numerical models of lattice structures.

(36) $$D_{s}=\frac{d_{real}-d_{CAD}}{d_{CAD}}$$

(37) $$k_{eff}^{real}=k_{eff}^{model}\left(1+\delta_{AM}\right)$$

(38) $$\delta_{AM}=f\left(D_{s},R_{a},P_{n}\right)$$

The sensitivity analysis performed in the present study indicates that strut-thickness deviation has the strongest influence on the effective thermal conductivity. This is consistent with previous studies showing that thickness variation and dimensional deviations are among the dominant geometry-related defects in lattice structures [88]. A ±10% change in strut diameter may lead to an approximately 6–10% variation in k_eff_, mainly due to changes in relative density and the solid-phase conduction contribution. Surface roughness primarily affects specific surface area, pore hydraulic diameter, and gas-phase tortuosity. Previous studies on additively manufactured lattice struts have shown that surface morphology depends strongly on strut diameter, inclination angle, and manufacturing conditions [89]. In the present sensitivity analysis, the influence of typical SLA surface roughness on total effective thermal conductivity was estimated to be below 4%.

Local microporosity or defects near nodal regions may reduce the effective conductivity of the solid phase and introduce additional uncertainty into the heat-transfer model. X-ray computed tomography has been widely used to identify and quantify such process-induced imperfections in lattice structures, including thickness variations, cross-sectional deviations, and nodal defects [90]. In the present model, nodal microporosity in the range of 1–5% was estimated to reduce k_eff_ by approximately 2–5%.

The obtained results indicate that manufacturing-induced imperfections are one of the main sources of discrepancy between mathematical predictions and experimental measurements. Future work should therefore include high-resolution micro-computed tomography reconstruction of the real lattice geometry, allowing manufacturing defects to be directly incorporated into STL-based thermal models, as shown in Table 5.

### 3.4. Convective Thermal Metamaterials: Opportunities, Limitations, and Future Perspectives

The majority of thermal metamaterial research has historically focused on the manipulation of conductive heat transfer. Pioneering studies by Narayana and Sato [91] demonstrated that engineered anisotropic thermal conductivity distributions could redirect heat flux around an object, giving rise to the concept of thermal cloaking. Shortly thereafter, Guenneau et al. [92] extended transformation thermodynamics to thermal concentrators and thermal rotators, showing that spatially varying conductivity tensors could focus or rotate heat flux trajectories. Experimental demonstrations were subsequently reported by Schittny et al. [93] and Han et al. [94], confirming the practical feasibility of transformation-based thermal devices.

More recently, attention has shifted toward convective thermal metamaterials, in which both fluid flow and heat transfer are manipulated simultaneously. Unlike purely conductive systems, convective thermal metamaterials exploit the coupling between permeability, hydraulic resistance, buoyancy-driven flow, and thermal transport. Li et al. [15] identified fluid–thermal coupling as one of the major future directions in thermal metamaterial research and highlighted the importance of architected porous structures capable of controlling both heat flux and fluid motion.

Several studies have investigated topology-dependent convection in porous and architected materials. Nield and Bejan [54] demonstrated that permeability, pore size, and tortuosity strongly influence the onset of natural convection through their effect on the Rayleigh number. Similarly, Vafai [55] showed that cellular architectures can suppress buoyancy-driven flow even under significant temperature gradients when pore connectivity and hydraulic diameter are appropriately controlled. These observations suggest that topology itself can act as a thermal design parameter independent of porosity.

Architected cellular metamaterials produced by additive manufacturing provide particularly attractive platforms for convective thermal management because their permeability and hydraulic diameter can be controlled independently through geometric design. Al-Ketan and Abu Al-Rub [16] demonstrated that TPMS-based cellular structures exhibit large variations in permeability despite similar relative densities. Comparable observations were reported by Maskery et al. [17] for gyroid architectures and by Yan et al. [95] for octet- and Kelvin-based lattices. These studies indicate that permeability engineering may be used to control fluid-assisted thermal transport without modifying the constituent material.

The present results support these findings. The proposed model predicts that the hydraulic diameter and topology-dependent convection suppression coefficient play a major role in determining the transition between conduction-dominated and convection-influenced transport regimes. Structures characterized by small hydraulic diameters and high tortuosity exhibit strong suppression of natural convection, whereas larger pore networks increase the likelihood of buoyancy-induced transport. Consequently, two cellular structures possessing identical porosity may exhibit substantially different effective thermal conductivities due to differences in permeability and flow resistance.

Recent developments have also introduced multifunctional metamaterials capable of simultaneously manipulating thermal, acoustic, mechanical, and fluid-flow properties. For example, Cheng et al. [96] proposed an interwoven dual-phase metamaterial architecture exhibiting coupled acoustic, mechanical, and transport functionalities. Zhang et al. [97] reported bioinspired architected metamaterials with enhanced energy absorption and flow-control capabilities. Similarly, non-integer-dimensional architected materials have been shown to enable simultaneous control of mechanical stiffness, permeability, and transport properties [98]. These studies suggest that future thermal metamaterials may evolve toward fully multifunctional systems integrating thermal insulation, heat exchange, fluid management, and mechanical load-bearing functions within a single architected material.

Despite these advances, several challenges remain. Accurate prediction of topology-dependent permeability, validation of local flow fields, manufacturing-induced geometric deviations, and the lack of experimentally validated homogenization frameworks continue to limit the practical implementation of convective thermal metamaterials. Furthermore, the majority of published studies focus on either conductive or fluid-flow behavior separately, whereas coupled conduction–convection modeling remains relatively underdeveloped.

Future research should therefore focus on topology-gradient cellular architectures, anisotropic permeability distributions, temperature-adaptive convective metamaterials, and AI-assisted inverse design frameworks capable of simultaneously optimizing thermal conductivity, permeability, and mechanical performance. The combination of additive manufacturing, computational homogenization, machine learning optimization, and high-resolution flow diagnostics offers a promising route toward next-generation fluid–thermal metamaterials with programmable transport properties.

## 4. Conclusions

The first major outcome of the presented synthesis is an engineering redefinition of the concept of thermal metamaterials in relation to cellular structures. The conducted analytical and experimental investigations demonstrated that architected cellular metamaterials fabricated using additive manufacturing technologies can provide widely tunable thermal-insulation properties controlled primarily by the topology of the cellular architecture and the geometry of the air cells (pores). The developed mathematical model successfully coupled heat conduction through the solid skeleton, gas-phase conduction inside the air cells, internal thermal radiation, and topology-dependent heat-transfer mechanisms. The proposed model demonstrated good predictive capability, with an average deviation of approximately 5.5–5.6% between theoretical predictions and experimental measurements. All investigated configurations remained within the 10% validation interval, confirming the applicability of the proposed homogenization-based framework for highly porous additively manufactured lattice metamaterials.

The obtained results demonstrated that porosity alone is insufficient to describe the effective thermal conductivity of cellular metamaterials. Instead, parameters such as hydraulic diameter, tortuosity, specific surface area, and the connectivity of the cellular network play a crucial role in governing heat transport within architected porous structures. The performed investigations additionally revealed the existence of a geometric transition region near L_c_ = 6 mm. Below this critical value, heat transfer was dominated primarily by conduction and thermal radiation, whereas larger cell sizes promoted local enhancement in gas motion induced by buoyancy forces due to the rapid increase in the effective Rayleigh number.

A limitation of the present study is that experimental validation was performed only within a relatively narrow temperature range centered around 0 °C. Although the proposed model includes a temperature-dependent radiative component, the influence of temperature-dependent material properties and high-temperature radiative transport was not experimentally investigated. Future work should therefore focus on extending the model toward wider temperature ranges, incorporating temperature-dependent thermal conductivity, emissivity, permeability, and radiative transport effects.

The experimental configuration corresponding to a horizontally oriented specimen with top-to-bottom heat flow produced gravitationally stable conditions, suppressing the development of large-scale natural convection. Consequently, heat transfer was governed predominantly by conductive and radiative mechanisms.

The obtained results confirmed that homogenization approaches based on STL geometry can successfully predict the thermal behavior of architected porous metamaterials and may constitute an effective design tool for lightweight thermal-insulation materials, multifunctional metamaterials, energy-efficient sandwich structures, and additively manufactured thermal-management systems.

From a biomimetic perspective, the investigated cellular metamaterials demonstrate how fundamental design principles observed in natural cellular systems can be transferred into engineered thermal-management structures. Rather than reproducing exact biological geometries, the proposed architectures mimic key functional characteristics of biological materials, including lightweight porous construction, hierarchical organization, optimized transport pathways, and topology-controlled thermal transport. The results confirm that biomimetic design provides an effective strategy for tailoring thermal conductivity in additively manufactured cellular metamaterials and may contribute to the development of next-generation multifunctional thermal insulation and thermal-management systems.

The present study assumes isotropic effective thermal conductivity and evaluates heat transfer only along one principal direction. Although this assumption is appropriate for comparative analysis and for highly symmetric lattice architectures, some investigated topologies may exhibit anisotropic thermal behavior. Future work should therefore focus on determining the complete thermal conductivity tensor through multidirectional experimental measurements and numerical homogenization procedures, enabling the design of anisotropic thermal metamaterials with programmable heat-flow characteristics.

## Figures and Tables

**Table 1:** Biological inspirations and biomimetic design principles underlying the investigated lattice-based cellular thermal metamaterials.

Lattice Topology	Biological Analogue	Biomimetic Principle	Key References
Kelvin	Cancellous (trabecular) bone	Efficient material distribution, lightweight cellular architecture	[36,44]
Diamond lattice	Trabecular bone network	Interconnected transport pathways, high stiffness-to-weight ratio	[19,41]
Gyroid (TPMS)	Biological membranes, butterfly wing nanostructures, cellular membranes	Continuous transport channels, large surface-to-volume ratio	[45,46,47]
Rhombic dodecahedron	Natural cellular foams, plant parenchyma	Nearly isotropic transport and load distribution	[8,19]
Tetrahedron-based	Radiolarians, diatoms	Hierarchical cellular organization, optimized load transfer	[41,48]
BCC	Porous skeletal systems, cancellous bone-inspired architectures	Optimized connectivity and material efficiency	[43]
FCC	Cellular biological frameworks	Enhanced nodal connectivity and multidirectional transport pathways	[43,49]
Octet Truss	Marine sponge skeletons (*Euplectella aspergillum*)	Stretch-dominated architecture, high specific stiffness	[50,51]
Iso-Truss	Bamboo vascular structure	Efficient load distribution and hierarchical reinforcement	[41,42]

**Table 2:** Comparison between numerical-model predictions and experimental results for the top–down heat-flow configuration.

$k_{panel_{model}}$ [W/mK]		L_c_			
**n**	ε [-]	4	6	8	10
1	0.9	0.0508	0.055	0.0592	0.0634
1	0.95	0.045	0.0492	0.0534	0.0576
2	0.9	0.047	0.0491	0.0512	0.0533
2	0.95	0.0412	0.0433	0.0454	0.0475
3	0.9	0.0458	0.0472	0.0486	0.05
3	0.95	0.04	0.0414	0.0428	0.0442
**k_exp approx_ [W/mK]**					
**n**	ε [-]	4	6	8	10
1	0.9	0.0467	0.0525	0.0562	0.059
1	0.95	0.0438	0.052	0.0573	0.062
2	0.9	0.0437	0.044	0.0478	0.049
2	0.95	0.0428	0.044	0.0455	0.0462
3	0.9	0.0421	0.041	0.043	0.0445
3	0.95	0.0388	0.0388	0.042	0.0443
**Δk _model-exp_ [W/mK]**					
**n**	ε [-]	4	6	8	10
1	0.9	0.0041	0.0025	0.003	0.0044
1	0.95	0.0012	−0.0028	−0.0039	−0.0044
2	0.9	0.0033	0.0051	0.0034	0.0043
2	0.95	−0.002	−0.0007	−0.0001	0.0013
3	0.9	0.0037	0.0062	0.0056	0.0055
3	0.95	0.0012	0.0026	0.0008	−0.0001
**Error [%]**					
**n**	ε [-]	4	6	8	10
1	0.9	8.8497	4.7919	5.3331	7.4188
1	0.95	2.8105	−5.3538	−6.8071	−7.1265
2	0.9	7.6134	11.663	7.1882	8.8557
2	0.95	−3.77	−1.6056	−0.2203	2.8252
3	0.9	8.8141	15.168	13.084	12.434
3	0.95	2.964	6.5974	1.8312	−0.2754

Here, n denotes the number of lattice layers forming the panel thickness, L_c_ is the characteristic unit-cell size, and ε is the porosity of the cellular structure.

**Table 3:** Comparison of modeling and experimental results for the bottom–up heat-flow configuration.

k_panel_model_ [W/mK]		L_c_			
**n**	ε [-]	4	6	8	10
1	0.9	0.0581	0.0622	0.0664	0.0706
1	0.95	0.0523	0.0564	0.0606	0.0648
2	0.9	0.0513	0.0534	0.0555	0.0576
2	0.95	0.0455	0.0476	0.0497	0.0518
3	0.9	0.0458	0.0472	0.0486	0.0500
3	0.95	0.0399	0.0414	0.0428	0.0442
**k_exp approx_ [W/mK]**					
**n**	ε [-]	4	6	8	10
1	0.9	0.0520	0.0585	0.0660	0.0673
1	0.95	0.0488	0.0578	0.0625	0.0655
2	0.9	0.0530	0.0520	0.0540	0.0588
2	0.95	0.0485	0.0530	0.0535	0.0545
3	0.9	0.0530	0.0545	0.0540	0.0532
3	0.95	0.0495	0.0525	0.0538	0.0536
**Δk _model-exp_ [W/mK]**					
**n**	ε [-]	4	6	8	10
1	0.9	0.0061	0.0037	0.0004	0.0033
1	0.95	0.0035	−0.0014	−0.0019	−0.0007
2	0.9	−0.0017	0.0014	0.0015	−0.0012
2	0.95	−0.0030	−0.0054	−0.0038	−0.0027
3	0.9	−0.0072	−0.0073	−0.0054	−0.0032
3	0.95	−0.0096	−0.0111	−0.0110	−0.0094
**Error [%]**					
**n**	ε [-]	4	6	8	10
1	0.9	11.6539	6.3938	0.6350	4.8981
1	0.95	7.0939	−2.3455	−2.9997	−1.0618
2	0.9	−3.1720	2.7348	2.8234	−1.996
2	0.95	−6.2212	−10.211	−7.1163	−4.9603
3	0.9	−13.565	−13.36	−9.9515	−5.9524
3	0.95	−19.293	−21.219	−20.504	−17.578

Here, n denotes the number of lattice layers forming the panel thickness, L_c_ is the characteristic unit-cell size, and ε is the porosity of the cellular structure.

**Table 4:** Comparison of experimental results and mathematical model predictions.

Structure	$k_{model}$	$k_{exp}$	Error [%]
All_face_cubic	0.045	0.043	4.65
Auxetic	0.043	0.047	8.51
BCC	0.041	0.039	5.13
BCCZ	0.042	0.044	4.55
CUBIC	0.044	0.042	4.76
Cuboctahedron Z	0.046	0.050	8.00
Diamond	0.042	0.040	5.00
FBCC	0.045	0.043	4.65
FBCCXYZ	0.052	0.050	4.00
FBCCZ	0.047	0.043	9.30
FCC	0.043	0.041	4.88
FCCZ	0.044	0.045	2.22
G7	0.045	0.046	2.17
Iso truss	0.048	0.046	4.35
Kelvin	0.044	0.046	4.35
Octahedron	0.042	0.045	6.67
Octet	0.048	0.051	5.88
Rhombic dodecahedron	0.045	0.043	4.65
Tetrahedron	0.057	0.053	7.55
Truncated cube	0.045	0.049	8.16
Truncated cuboctahedron	0.047	0.050	6.00

**Table 5:** Sensitivity analysis of the influence of representative SLA manufacturing defects on the effective thermal conductivity of cellular thermal metamaterials.

Defect Type	Assumed Defect Magnitude	Change in k_eff_
Strut thickness variation	±10%	6–10%
Surface roughness	Ra = 5–20 μm	1–4%
Nodal microporosity	1–5%	2–5%

## Data Availability

The original contributions presented in this study are included in the article/Supplementary Material. Further inquiries can be directed to the corresponding author.

## Supplementary Materials

The following supporting information can be downloaded at: https://www.mdpi.com/article/10.3390/ma19142992/s1, Sections S1–S15: Extended Mathematical Model for Topology—Controlled Heat Transfer in Open-Cell Thermal Metamaterials; Table S1: Comparison of the mathematical-model predictions for the investigated lattice structures. Refs. [99,100,101,102,103,104,105,106,107,108,109,110,111,112,113,114,115,116,117,118,119,120] can be found in Supplementary Materials.

## Abbreviations

The following abbreviations are used in this manuscript:

V_s_	skeleton volume
V_cell_	bounding-cell volume
ρ_r_	relative density
ε	porosity
A_s_	skeleton surface area
S_v_	specific surface area
D_h_	hydraulic pore diameter
A_in.f_	effective inlet area
ϵ_A_	surface porosity
τ_g_	tortuosity
K	permeability
Nu	Nusselt number
Re	pore Reynolds number
Pe	Peclet number
