# Supplementary material for: Additively Manufactured Bionic Cellular Metamaterials with Controllable Thermal Conductivity—Mathematical Models and Experimental Research
Source: Materials (Basel). 2026 Jul 10;19(14):2992. doi: 10.3390/ma19142992 (PMC13412845; doi:10.3390/ma19142992)
Supplement: Supplementary file 1 [file materials-19-02992-s001.zip › materials-4344409-supplementary.pdf]

Section S1–S15. Extended Mathematical Model for Topology-Controlled Heat Transfer in Open-Cell Thermal Metamaterials

S1. Mathematical Model of Heat Transfer in Cellular Structures

Recent studies have demonstrated that the thermal behavior of architected cellular materials is governed not only by porosity and relative density but also by the directional arrangement of the structural network [1–4]. Consequently, many periodic lattice architectures exhibit anisotropic effective thermal conductivity, even when manufactured from isotropic constituent materials [2,5].

In anisotropic media, thermal conductivity is represented by a second-order tensor [1,2]
Fourier's law for an anisotropic material

$$q = -K\nabla T \quad \text{(S1)}$$

or, in component form:

$$q_i = -\frac{K_{ij}(\partial T)}{\partial x_j} \quad \text{(S2)}$$

thermal conductivity tensor

$$K = \begin{matrix} k_{xx} & k_{xy} & k_{xz} \\ k_{yx} & k_{yy} & k_{yz} \\ k_{zx} & k_{zy} & k_{zz} \end{matrix} \quad \text{(S3)}$$

Orthotropic case
For most lattice structures:

$$K = \begin{matrix} k_{xx} & 0 & 0 \\ 0 & k_{yy} & 0 \\ 0 & 0 & k_{zz} \end{matrix} \quad \text{(S4)}$$

Assumption adopted in the present manuscript:

$$K = k_{(eff)} I \quad \text{(S5)}$$

where:

$$I = \begin{matrix} 1 & 0 & 0 \\ 0 & 1 & 0 \\ 0 & 0 & 1 \end{matrix} \quad \text{(S6)}$$

in other words:

$$K = \begin{matrix} k_{eff} & 0 & 0 \\ 0 & k_{eff} & 0 \\ 0 & 0 & k_{eff} \end{matrix} \quad \text{(S7)}$$

Tensor transformation under coordinate rotation

$$K' = RKR^T$$

Where:
R - denotes the rotation matrix

In the most general case, the effective thermal conductivity of a lattice metamaterial is tensorial. However, since the present experimental validation was performed along a single principal heat-flow direction, the model was reduced to a scalar effective conductivity corresponding to the investigated measurement direction. This reduction is appropriate for the comparative analysis performed in this study, but it does not represent a full tensor characterization of the investigated architectures.

Principal thermal conductivity values

$$K = \begin{matrix} k_1 & 0 & 0 \\ 0 & k_2 & 0 \\ 0 & 0 & k_3 \end{matrix} \quad \text{(S8)}$$

or:

$$K = \begin{matrix} k_I & 0 & 0 \\ 0 & k_{II} & 0 \\ 0 & 0 & k_{III} \end{matrix} \quad \text{(S9)}$$

Anisotropy ratio

$$A_k = \frac{k_{max}}{k_{min}} \tag{S10}$$

Anisotropy ratios for principal directions

For comparison between directions:

$$A_{xy} = \frac{k_{xx}}{k_{yy}} \tag{S11}$$

$$A_{xz} = \frac{k_{xx}}{k_{zz}} \tag{S12}$$

$$A_{yz} = \frac{k_{yy}}{k_{zz}} \tag{S13}$$

The condition of isotropy

$$k_{xx} = k_{yy} = k_{zz} \tag{S14}$$

then: $A_k = 1$

Anisotropy condition

$$k_{xx} \neq k_{yy} \neq k_{zz} \tag{S15}$$

or:

$$A_k > 1$$

Equation proposed for the future experimental validation plan

For measurements performed in three directions:

$$K_{exp} = \begin{matrix} k_x & 0 & 0 \\ 0 & k_y & 0 \\ 0 & 0 & k_z \end{matrix} \tag{S16}$$

and :

$$A_k = \frac{\max(k_x, k_y, k_z)}{\min(k_x, k_y, k_z)} \tag{S17}$$

The cellular metamaterial considered in this study is treated as a heterogeneous medium composed of two phases [6–8]:

- a solid phase (Y_s) forming the structural skeleton,
- a porous phase (Y_p) or fluid phase (Y_f), filled with gas or fluid.

At the macroscopic scale, the material occupies the domain:

$$\Omega \subset R^d. \ d = 2 \ lub \ 3 \ (1) \tag{S18}$$

whereas at the microscale its internal architecture is described by a representative volume element (RVE):

$$Y = [0.1]^d \tag{S19}$$

A small-scale parameter is introduced [6,9,10]:

$$\varepsilon_h = \frac{\ell}{L}. \ 0 < \varepsilon_h \ll 1 \tag{S20}$$

where:

ℓ - denotes the characteristic cell size,

L - denotes the characteristic dimension of the panel or specimen.

Porosity and relative density are defined as [1,6]:

$$\varepsilon = \frac{|Y_p|}{|Y|} \tag{S21}$$

$$\frac{\rho *}{\rho_s} = \frac{|Y_S|}{|Y|} = 1 - \varepsilon \tag{S22}$$

where:

ε - porosity,

ρ^* - effective density of the cellular material,

ρ_s - density of the bulk solid material.

The model distinguishes between two topological classes of cellular structures: closed-cell and open-cell systems. The mathematical formulation for closed-cell structures was previously developed by the author in Ref. [11].

In contrast, open-cell structures contain interconnected pore networks, enabling not only conduction through the solid skeleton, but also heat transfer through the gaseous phase, internal thermal radiation, and fluid or gas flow associated with convective–advective transport mechanisms [12–15].

It is precisely this second class of structures that requires, within the scope of the present study, an extension of the classical heat-transfer model.

S2. Local Heat-Conduction Problem in a Heterogeneous Medium

Under steady-state conditions and in the absence of volumetric heat sources, the local heat-conduction equation takes the following form [6–8]:

$$\nabla \cdot \left(k\left(\frac{x}{\varepsilon_h}\right) \nabla T^{\{\varepsilon_h\}(x)} \right) = 0 \tag{S23}$$

where:

$T^{\{\varepsilon_h\}(x)}$ — denotes the local temperature field,

$k\left(\frac{x}{\varepsilon_h}\right)$ — is the local thermal conductivity coefficient dependent on the microstructure

Within the phase-based formulation [6,7,16]:

$$k(y) = k_s \;\; w \, Y_s \tag{S24}$$

$$k(y) = k_p \;\; w \, Y_p \tag{S25}$$

For open-cell structures containing gas-filled interconnected pores, it is more convenient to express the formulation as:

$$k(y) = k_f \;\; w \, Y_f \tag{S26}$$

At the solid–fluid interface $\Gamma_{\{sf\}}$ ideal thermal-contact conditions are imposed:

$$T_s = T_f \tag{S27}$$

$$-\frac{k_s \partial T_s}{\partial n} = -\frac{k_f \partial T_f}{\partial n} \tag{S28}$$

which represent continuity of temperature and continuity of the normal heat flux across the interface.

S3. Variational Formulation

The heat-conduction equation may be expressed in weak form [7,8]. The objective is to determine:

$$T^{\{\varepsilon_h\}} \in H^{1(\Omega)} \tag{S29}$$

such that for an arbitrary test function: $v \backslash in\, H^{1(\Omega)}$, the following condition is satisfied:

$$\int_\Omega k\left(\frac{x}{\varepsilon_h}\right) \nabla T^{\{\varepsilon_h\}} \cdot \nabla v \, dx = 0 \tag{S30}$$

This formulation is equivalent to the stationarity condition of the energy functional:

$$J[T] = \left(\frac{1}{2}\right) \int_\Omega k\left(\frac{x}{\varepsilon_h}\right) |\nabla T|^2 dx \tag{S31}$$

namely:

$$\delta J[T] = 0 \tag{S32}$$

S4. Two-Scale Homogenization

A fast microscale variable is introduced [6–8]:

$$y = \frac{x}{\varepsilon_h} \tag{S33}$$

together with the decomposition of the gradient operator:

$$\nabla = \nabla_x + \left(\frac{1}{\varepsilon_h}\right) \nabla_y \tag{S34}$$

The temperature field is asymptotically expanded as:

$$T^{\{\varepsilon_h\}(x)} = T_{0(x.y)} + \varepsilon_h T_{1(x.y)} + \varepsilon_h^2 T_{2(x.y)} + \ldots \tag{S35}$$

After substitution into the local governing equation and grouping terms according to powers of, a hierarchy of problems of successive orders is obtained.

rząd $\varepsilon_h^{\{-2\}}$

$$\nabla_y \cdot \left(k(y)\nabla_y T_0\right) = 0 \tag{S35}$$

For periodic boundary conditions, this yields:

$$T_{0(x.y)} = \bar{T}(x) \tag{S36}$$

indicating that the leading-order temperature component depends only on the macroscale.

rząd $\varepsilon_h^{\{-1\}}$

The corrector function is introduced in the form [6–8]:

$$T_{1(x.y)} = \frac{\Sigma_j \chi_{j(y)\partial\bar{T}}}{\partial x_j} \tag{S37}$$

Substitution leads to the cell problem:

$$\nabla_y \cdot \left(k(y)\left(e_j + \nabla_y \chi_j\right)\right) = 0 \tag{S38}$$

where: χ_j are correction functions describing the influence of the microgeometry on macroscopic heat transport.

The effective thermal conductivity is finally described by the homogenized tensor:

$$k_{eff.ij} = \left(\frac{1}{|Y|}\right) \int_Y k(y) \left(\delta_{ij} + \frac{\partial \chi_j}{\partial y_i}\right) dY \tag{S39}$$

and the macroscopic governing equation becomes:

$$\nabla \cdot \left(K_{eff} \nabla \bar{T}\right) = 0 \tag{S240}$$

For isotropic systems, the tensor reduces to:

$$K_{eff} = k_{eff} I \tag{S41}$$

For open-cell structures, the model must be extended to include heat conduction through the gas phase occupying the interconnected pores [12–15,17,18].

Consequently, the proposed final model is expressed as::

$$k_{eff,STL}^{op} = k_{cond,s} + k_{gas} + k_{conv,eff} + k_{rad,STL} \tag{S42}$$

that is [19–23]:

$$k_{eff,STL}^{op} = k_{cond,s} + Nu_{eff}\left(\frac{\varepsilon}{\tau_g}\right) k_g + k_{rad,STL} \tag{S43}$$

S5. Conduction Through the Solid Skeleton

For strut-based open-cell structures [1,20,24]:

$$k_{cond.s} = k_s \left[\left(\frac{1}{3}\right) \rho_r + \left(\frac{2}{3}\right) \rho_r^{\frac{3}{2}} \right] \tag{S44}$$

The high-porosity Lemlich-type limit provides the simplified approximation:

$$k_{cond.s} \approx \frac{k_s(1-\varepsilon)}{3} \tag{S45}$$

which may be treated as a reference model.

S6. Gas-Phase Conduction

In open-cell structures, the gaseous phase forms a continuous interconnected network. The gas contribution is expressed as [17–19]:

$$k_{cond,g} = \left(\frac{\varepsilon}{\tau_g}\right) k_g \tag{S46}$$

where

τ_g - tortuosity coefficient,.

k_g - thermal conductivity of the gas..

As a first approximation [13–15]:

$$\tau_g = 1 \tag{S47}$$

thus:

$$k_{cond,g} \approx \varepsilon\, k_g \tag{S48}$$

S7. Thermal Radiation

$$k_{rad} = 4\, \varepsilon_{rad} \sigma\, T_m^3 L_r \tag{S49}$$

In open-cell structures: L_r does not necessarily correspond to a single pore dimension, but may instead be related to the effective radiative view path within the interconnected pore network.

$$T_m = \frac{T_{hot}+T_{cold}}{2} \tag{S50}$$

$$L_r = \chi_r D_h \tag{S51}$$

$$\chi_r \approx 0.5 \tag{S52}$$

S8. Local and Panel-Scale Convection [25]

Cell Volume and Skeleton Volume:

$$V_{cell} = L_x * L_y * L_z \tag{S53}$$

For a cubic unit cell:

$$L_x = L_y = L_z = L_c \tag{S54}$$

therefore:

$$V_{cell} = L_c^3 \tag{S55}$$

Solid and pore volumes

$$V_s = V_{STL} \tag{S56}$$

$$V_p = V_{cell} - V_s \tag{S57}$$

where:
V_{cell} - denotes the volume of the STL bounding cell,
V_s - denotes the volume of the solid material extracted from the STL geometry,
V_p - denotes the pore volume.

Relative Density and Porosity:

$$\rho_r = \frac{V_s}{V_{cell}} \tag{S58}$$

thus:

$$V_p = \varepsilon V_{cell} \tag{S59}$$

For a cubic unit cell:

$$V_p = \varepsilon L_c^3 \tag{S60}$$

$$\varepsilon = \frac{V_p}{V_{cell}} = 1 - \rho_r \tag{S61}$$

Skeleton Surface Area and Specific Surface Area
The total surface area of the triangulated STL mesh is determined as:

$$A_s = \sum(A_{triangle}) \tag{S62}$$

The specific surface area is then defined as:

$$S_v = \frac{A_s}{V_{cell}} \tag{S63}$$

where: Sv describes the ratio of the solid–gas interfacial area to the total cell volume.
therefore:

$$A_s = S_v V_{cell} \tag{S64}$$

For a cubic unit cell:

$$A_s = S_v L_c^3 \tag{S65}$$

Effective Hydraulic Diameter of the Pores

For open-cell structures:

$$D_h = \frac{4 * V_p}{A_s} \tag{S66}$$

Substituting $V_p = \varepsilon V_{cell}$ and $A_s = S_v V_{cell}$:

$$D_h = \frac{4\varepsilon V_{cell}}{S_v V_{cell}} \tag{S67}$$

After simplification:

$$D_h = \frac{4 * \varepsilon}{S_v} \tag{S69}$$

This expression shows that D_h is directly related to porosity and specific surface area.

Scaling with nominal cell size

For geometrically similar lattice cells, the specific surface area scales approximately as:

$$S_v = \frac{C_s}{L_c} \tag{S70}$$

where C_s is a topology-dependent dimensionless surface coefficient.

Substituting this relation into:

$$D_h = \frac{4\varepsilon}{S_v} \tag{S71}$$

gives:

$$D_h = \frac{4\varepsilon}{\frac{C_s}{L_c}} \tag{S72}$$

therefore:

$$D_h = \frac{4\varepsilon L_c}{C_s} \tag{S73}$$

The above relation may be written as:

$$D_h = C_D\, \varepsilon L_c \tag{S74}$$

where:

$$C_D = \frac{4}{C_s} \tag{S75}$$

is a topology-dependent hydraulic coefficient.

<u>Replacement of the Nominal Cell Size by the Hydraulic Diameter in the Rayleigh Number Formulation</u>

The Rayleigh number initially written with the nominal cell size is:

$$Ra_c = \frac{g\beta\Delta T\, L_c^3}{\nu\alpha_g} \tag{S76}$$

where:

β - volumetric thermal-expansion coefficient,.

ν - kinematic viscosity.

α_g - thermal diffusivity of the gas.

L_c - characteristic cell size,.

H - panel thickness..

However, for open-cell structures, the hydraulic diameter is the more physically meaningful characteristic length. Therefore, the Rayleigh number should be written as:

$$Ra_{D_h} = \frac{g\beta\Delta T\, D_h^3}{\nu\alpha_g} \tag{S77}$$

After substituting:

$$D_h = C_D\, \varepsilon L_c \tag{S78}$$

we obtain:

$$Ra_{D_h} = \frac{g\beta\Delta T\, (C_D \varepsilon L_c)^3}{\nu\alpha_g} \tag{S79}$$

that is:

$$Ra_{D_h} = C_D^3 \varepsilon^3 \frac{g\beta\Delta T\, L_c^3}{\nu\alpha_g} \tag{S80}$$

Since:

$$Ra_c = \frac{g\beta\Delta T\, L_c^3}{\nu\alpha_g} \tag{S81}$$

the relationship between both Rayleigh numbers becomes:

$$Ra_{D_h} = C_D^3\, \varepsilon^3 Ra_c \tag{S82}$$

This formulation formally links the nominal cell size L_c with the hydraulic diameter D_h and demonstrates that the effective buoyancy-driven transport in open-cell lattices depends not only on cell size, but also on porosity and topology.

In all mathematical calculations presented in this study, the Rayleigh number was evaluated using the hydraulic diameter D_h extracted directly from the STL geometry rather than the nominal cell size L_c.

Final interpretation

The nominal cell size L_c is useful for describing the external geometry of the lattice. However, the hydraulic diameter D_h should be used in the convective part of the model because it accounts for the actual pore volume, solid–gas interfacial area, and topology-dependent flow constraints.

Therefore, in the final form of the model, the characteristic length in the Rayleigh number is replaced as:

$$L_c \to D_h \quad \text{(S83)}$$

and the convective contribution is evaluated using:

$$Ra_{D_h} = \frac{g\beta\Delta T\, D_h^3}{\nu\alpha_g} \quad \text{(S84)}$$

instead of:

$$Ra_c = \frac{g\beta\Delta T\, L_c^3}{\nu\alpha_g} \quad \text{(S85)}$$

This resolves the inconsistency between the nominal geometric description of the lattice and the physically relevant transport length scale governing convection in open-cell cellular metamaterials.

$$Nu_{eff} = 1 + \eta_{top}\eta_w\left(Nu_{D_h} - 1\right) \quad \text{(S86)}$$

where:

η_{op}- topological suppression coefficient,

η_w - suppression coefficient associated with layering and orientatio.

Nu— Nusselt number determined from geometry and flow conditions.

Nu=1 oznacza samo przewodzenie gazowe.

Conduction + Convection

$$k_{gas+conv} = Nu_{eff}\left(\frac{\varepsilon}{\tau_g}\right)k_g \quad \text{(S87)}$$

The convection element itself

$$k_{conv,eff} = (Nu_{eff} - 1)\left(\frac{\varepsilon}{\tau_g}\right)k_g \quad \text{(S88)}$$

S9. Flow Model in Open-Cell Structures

If the air cells (pores) are interconnected, the fluid-flow problem must be solved explicitly within the fluid domain [14,15]: Continuity equation:

$$\frac{\partial}{\partial x_i(\rho\, u_i)} = 0 \quad \text{(S89)}$$

For incompressible flow:

$$\frac{\partial u_i}{\partial x_i} = 0 \quad \text{(S90)}$$

Pressure decomposition:

$$\frac{\partial P}{\partial x_i} = -\left(\frac{\partial \bar{p}}{\partial x_i}\right)e_{L.i} + \frac{\partial \hat{p}}{\partial x_i} \quad \text{(S91)}$$

where::

p̄ — macroscopic pressure drop.

p̂ — periodic component

Momentum equation:

$$\frac{\partial}{\partial x_j(\rho\, u_j u_i)} = -\frac{\partial \hat{p}}{\partial x_i} + \frac{\partial}{\partial x_j\left(\mu\frac{\partial u_i}{\partial x_j}\right)} - \left(\frac{\partial \bar{p}}{\partial x_i}\right)e_{L.i} \quad \text{(S92)}$$

Energy equation in the fluid phase:

$$\frac{\partial}{\partial x_i\left[(\rho\, C_p)_f u_i T_f\right]} = \frac{\partial}{\partial x_i\left(\frac{k_f \partial T_f}{\partial x_i}\right)} \quad \text{(S93)}$$

This formulation formally introduces advection into the thermal problem.

S10. Periodic Conditions and Bulk Temperature

Periodic conditions are imposed within the periodic module [10,11]:

$$u_{i(r)} = u_{i(r+\vec{L})} \tag{S94}$$

$$T(r) - T_{b(r)} = T\left(r + \vec{L}\right) - T_{b(r+\vec{L})} \tag{S95}$$

The bulk temperature is defined as:

$$T_b = \frac{\left(\int_A u_i e_{L.i} T dA\right)}{\left(\int_A u_i e_{L.i} dA\right)} \tag{S96}$$

This quantity is required for the definition of the Nusselt number and the heat-transfer coefficient.

S11. Nusselt Number, Permeability and Hydraulic Resistance

The Nusselt number is defined as [15,19,26]:

$$Nu = \frac{h\,D}{k_f} = \frac{q''D}{\left[k_f(T_s - T_b)\right]} \tag{S97}$$

Darcy permeability:

$$K = -\mu \frac{u_{mean}}{\left(\frac{\partial \bar{p}}{\partial x_i}\right)} \tag{S98}$$

Modified Reynolds number:

$$Re_K = Re_d\left(\frac{\sqrt{K}}{D}\right) \tag{S99}$$

Friction factor:

$$f = \frac{\left[\left(-\frac{\partial \bar{p}}{\partial x_i}\right)\sqrt{K}\right]}{\left[\rho\, u_{mean}^2\right]} \tag{S100}$$

In the Darcy regime:

$$f \approx \frac{1}{Re_K} \tag{S101}$$

or according to experimental correlations:

$$f \approx 0.057 + \frac{1}{Re_K} \tag{S102}$$

$$f \approx 0.105 + \frac{1}{Re_K} \tag{S103}$$

This formulation enables the effective convection to be related directly to the actual flow topology.

S12. Peclet Number

According to the literature [15,19], the Nusselt number scales well with:

$$\left(\frac{Pe_d}{1-\varepsilon}\right)^{\frac{1}{2}} \tag{S104}$$

where [15]:

$$Pe_d = Re_d Pr \tag{S105}$$

Thus, the following semi-empirical closure may be adopted [15,19]:

$$Nu = \Phi\left(\left(\frac{Pe_d}{1-\varepsilon}\right)^{\frac{1}{2}}.topology\right) \tag{S106}$$

allowing different skeletal structures to be distinguished for different porosity levels

For the skeletal model, explicit geometrical relationships are provided as follows.

The volume of the intersection between two spheres is given by [25]:

$$V_{int} = \frac{\pi}{12}(4R + s)(2R - s)^2 \tag{S107}$$

where:

R- is the radius of the sphere,

s - is the center-to-center distance between the in-line spheres.

The corrected volume of the central sphere is expressed as [25]:

$$V'_{bc} = \frac{4\pi}{3}R^3 - 8\left(\frac{V_{int}}{2}\right) \quad \text{(S108)}$$

The volume of the spherical cap is given by [50]:

$$V'_{sc} = \frac{\pi}{3}\left(R - \frac{s}{2}\right)^2\left(2R + \frac{s}{2}\right) \quad \text{(S109)}$$

The porosity is defined as [25]:

$$\varepsilon = \frac{V'_f}{a^3} \quad \text{(S110)}$$

The fluid inlet area is expressed as [[25]:

$$A_{in.f} = \pi R^2 - 2\left[2R^2\cos^{-1}\left(\frac{s}{2R}\right) - \left(\frac{1}{2}\right)s\sqrt{4R^2 - s^2}\right] + \pi\frac{4R^2 - a^2}{4} \quad \text{(S111)}$$

These relationships enable the transition from purely geometrical parameters to porosity, flow area, effective pore diameter, and the transport descriptors Nu, K [15,25].

S13. Hashin–Shtrikman Bounds

Every effective-medium model must satisfy admissibility constraints [16]:

$$k^{+}_{HS} = k_s + \frac{1-\varepsilon}{\frac{1}{k_p - k_s} + \frac{\varepsilon}{3k_s}} \quad \text{(S112)}$$

and, in the limiting case: k_p to 0 the following condition must hold:

$$\left(\frac{k_{eff}}{k_s}\right) \leq \frac{1-\varepsilon}{1+\frac{\varepsilon}{2}} \quad \text{(S113)}$$

This criterion verifies whether the model overestimates thermal conductivity in a formally inadmissible manner.

The Hashin–Shtrikman bounds were used only to verify the admissibility of the conductive part of the effective thermal conductivity. They should not be applied directly to the total effective conductivity when radiative and convective contributions are included.

S14.. Temperature-Dependent Contribution Analysis of Heat-Transfer Mechanisms

If the model is extended to transient heat transfer, the governing equation becomes:

$$\rho^* c_{p.eff}\frac{\partial T}{\partial t} = \nabla \cdot \left(k_{eff}\nabla T\right) \quad \text{(S114)}$$

In the non-Fourier formulation:

$$\frac{\tau_q \partial q}{\partial t} + q = -k_{eff}\nabla T \quad \text{(S115)}$$

which leads to:

$$\tau_q \rho^* c_{p.eff}\frac{\partial^2 T}{\partial t^2} + \rho^* c_{p.eff}\frac{\partial T}{\partial t} = \nabla \cdot \left(k_{eff}\nabla T\right) \quad \text{(S116)}$$

In order to quantify the relative contribution of each heat-transfer mechanism, the total effective thermal conductivity of an open-cell lattice structure is decomposed into four components:

$$k^{(op)}_{eff(T_m)} = k_{(cond,s)(T_m)} + k_{(gas)(T_m)} + k_{(conv,eff)(T_m)} + k_{(rad)(T_m)} \quad \text{(S117)}$$

where T_m is the mean absolute temperature expressed in kelvins.

The solid-conduction contribution is calculated as:

$$k_{(cond,s)(T_m)} = k_{s(T_m)}[\left(\frac{1}{3}\right)\rho_r + \left(\frac{2}{3}\right)\rho_r^{\frac{3}{2}}] \quad \text{(S118)}$$

The gas-conduction contribution is determined from:

$$k_{(gas)(T_m)} = \left(\frac{\varepsilon}{\tau_g}\right)k_{g(T_m)} \quad \text{(S119)}$$

The effective convective contribution is expressed as:

$$k_{(conv,eff)(T_m)} = \left[Nu_{(eff)(T_m)} - 1\right]\left(\frac{\varepsilon}{\tau_g}\right)k_{g(T_m)} \quad \text{(S120)}$$

where:

$$Nu_{(eff)(T_m)} = 1 + \eta_{top}\eta_w[Nu_{(D_h)(T_m)} - 1] \quad \text{(S121)}$$

The radiation contribution is calculated using the linearized radiation model [1,24,27]:

$$k_{(rad)(T_m)} = 4\varepsilon_{rad}\sigma T_m^3 L_{r,STL} \quad \text{(S122)}$$

This relation shows that the radiative component depends on the third power of the absolute mean temperature. Therefore, the radiation contribution increases rapidly with increasing temperature. For

two different mean temperatures $T_{m,1}$ and $T_{m,2}$, assuming constant emissivity and radiative path length, the ratio of the radiative contributions is:

$$\frac{k_{(rad)(T_{m,2})}}{k_{(rad)(T_{m,1})}} = \left(\frac{T_{m,2}}{T_{m,1}}\right)^3 \tag{S123}$$

For larger temperature differences, the more general linearized radiative heat-transfer coefficient may be used [28]:

$$h_{rad} = \varepsilon_{rad}\sigma(T_{hot}^2 + T_{cold}^2)(T_{hot} + T_{cold}) \tag{S124}$$

and therefore:

$$k_{rad} = h_{rad}L_{r,STL} \tag{S125}$$

The percentage contribution of each mechanism to the total effective thermal conductivity is then calculated as:

$$C_{cond,s} = \left(\frac{k_{cond,s}}{k_{eff}^{op}}\right) * 100\% \tag{S126}$$

$$C_{gas} = \left(\frac{k_{gas}}{k_{eff}^{op}}\right) * 100\% \tag{S127}$$

$$C_{conv} = \left(\frac{k_{conv,eff}}{k_{eff}^{op}}\right) * 100\% \tag{S128}$$

$$C_{rad} = \left(\frac{k_{rad}}{k_{eff}^{op}}\right) * 100\% \tag{S129}$$

where:

$$C_{cond,s} + C_{gas} + C_{conv} + C_{rad} = 100\% \tag{S130}$$

In the present experimental configuration, corresponding to top-to-bottom heat transfer, buoyancy-driven convection is strongly suppressed and therefore:

$$Nu_{eff} \approx 1$$

As a result:

$$k_{conv,eff} \approx 0$$

and the total heat transfer is governed mainly by solid conduction, gas conduction, and thermal radiation [29]

S15. Transient Formulation

After all model components have been included, the final formulation for open-cell structures is:

$$k_{eff,STL}^{op} = k_{cond,s} + k_{gas} + k_{conv,eff} + k_{rad,STL} \tag{S131}$$

that is:

$$k_{eff,STL}^{op} = k_s[\left(\frac{1}{3}\right)\rho_r + \left(\frac{2}{3}\right)\rho_r^{\frac{3}{2}}] + Nu_{eff}\left(\frac{\varepsilon}{\tau_g}\right)k_g + 4\varepsilon_{rad}\sigma T_m^3 L_{r,STL} \tag{S132}$$

<u>Effective Pore Inlet Area</u>

For a heat-flow direction aligned, for example, with the z-axis:

$$A_{face} = L_x * L_y \tag{S133}$$

$$A_{in.f} = A_{face} - A_{solid.cut} \tag{S134}$$

where: $A_{solid..cut}$ is the intersection area between the STL skeleton and the inlet plane.

In simplified form:

$$A_{inf.} \approx \varepsilon_A * A_{face} \tag{S135}$$

<u>Gas-Phase Tortuosity</u>

Tortuosity describes the elongation of the gas-flow path relative to the straight panel thickness:

$$\tau_g = \frac{L_{path}}{H} \tag{S136}$$

For a simplified STL-based approximation:

$$\tau_g \approx \frac{1}{\varepsilon_A} \quad \text{(S137)}$$

or more conservatively:

$$\tau_g \approx = \varepsilon^{-m} \quad \text{(S138)}$$

where: $m = 0.5 \ldots 1.0$

Gas-Phase Conduction in the Pores

The previous simplified model:

$$k_{g.eff} = \varepsilon * k_g \quad \text{(S139)}$$

should be replaced by the geometrically corrected formulation:

$$k_{g.eff} = \left(\frac{\varepsilon}{\tau_g}\right) * k_g \quad \text{(S140)}$$

indicating that gas-phase conduction decreases as the flow path becomes increasingly tortuous.

Permeability of Open-Cell Structures

The Kozeny–Carman model may be adopted:

$$K = \frac{\varepsilon^3}{(C_K * S_v^2)} \quad \text{(S141)}$$

where typically: $C_K = 4 \ldots 6$

Alternatively, using the hydraulic diameter:

$$K = C_{KD} * \varepsilon * D_h^2 \quad \text{(S142)}$$

where: C_{KD} is a topology-dependent coefficient.

Pore Reynolds Number

$$Re_d = \rho_f * u_{mean} * \frac{D_h}{\mu} \quad \text{(S143)}$$

Peclet Number

$$Pe_d = Re_d * Pr \quad \text{(S144)}$$

Nusselt Number for Open-Cell Structures

According to the referenced literature, the Nusselt number scales with:

$$Nu = F\left(\left(\frac{Pe_d}{(1-\varepsilon)}\right)^{\frac{1}{2}}.topology\right) \quad \text{(S145)}$$

In a semi-empirical form:

$$Nu = C_{Nu} * \left(\frac{Pe_d}{(1-\varepsilon)}\right)^n \quad \text{(S146)}$$

where typically: $n \approx 0.5$

Convective Contribution

Natural convection occurring inside open-cell lattice structures is strongly influenced by geometric constraints imposed by the cellular architecture. Unlike conventional fluid cavities, interconnected pore networks introduce additional resistance to buoyancy-driven flow through tortuous pathways, local constrictions, dead-end pores, and topology-dependent permeability variations [29,30]. To account for these effects, a topology-dependent suppression coefficient η_{top} is introduced:

$$Nu_{eff} = 1 + \eta_{top}\eta_w\left(Nu_{D_h} - 1\right) \quad \text{(S147)}$$

where:

η_{top}- topological suppression coefficient (describes the reduction of convective heat transfer caused by geometric flow restrictions within the lattice architecture),

η_w - suppression coefficient associated with layering and orientatio.

Nu— Nusselt number determined from geometry and flow conditions. Nu=1.

The coefficient depends primarily on pore connectivity, tortuosity, hydraulic diameter, permeability, strut arrangement, degree of branching [17,31–33]. Highly interconnected architectures exhibiting relatively straight flow paths are expected to produce larger values of η_{top}, whereas complex cellular networks characterized by highly tortuous pathways generate stronger suppression of natural

Convection [25,29]. Based on geometric considerations and previous studies of flow in porous media, the following indicative ranges may be adopted [19,29]:

$$0.7 \le \eta_{top} \le 1.0$$

for highly permeable lattices (BCC, FCC, open truss structures),

$$0.4 \le \eta_{top} \le 0.7$$

for intermediate topologies (Kelvin, Rhombic Dodecahedron),

and

$$0.2 \le \eta_{top} \le 0.5$$

for highly tortuous TPMS-based and complex cellular architectures.

A second correction factor η_w is introduced to account for finite-size effects and boundary-induced suppression of flow:

$$0.7 \le \eta_w \le 1.0$$

The coefficient represents the influence of specimen thickness, external boundaries, and thermal boundary conditions on the development of buoyancy-driven recirculation [29,30].

Both coefficients should be interpreted as semi-empirical correction factors rather than universal physical constants [25]. Their rigorous determination requires direct flow visualization experiments, permeability measurements, or CFD-based inverse calibration procedures. For the experimentally validated top-to-bottom heat-flow configuration, buoyancy-driven convection is strongly suppressed due to gravitational stability [29].

Future work will focus on determining η_{top} and η_w through Particle Image Velocimetry (PIV), numerical fluid-flow simulations, and permeability characterization of additively manufactured lattice structures.

$$k_{gas+conv} = Nu_{eff}\left(\frac{\varepsilon}{\tau_g}\right)k_g \quad \text{(S148)}$$

$$k_{conv,eff} = (Nu_{eff} - 1)\left(\frac{\varepsilon}{\tau_g}\right)k_g \quad \text{(S149)}$$

<u>Radiation with STL Geometrical Correction [27]</u>

Instead of the simplified relation:

$$k_{rad} = 4\varepsilon_{rad}\sigma T_m^3 L_r \quad \text{(S150)}$$

the following corrected form should be use:

$$k_{rad.STL} = 4 * \varepsilon_{rad} * \sigma * T_m^3 * L_{rSTL}. \quad \text{(S151)}$$

In this equation, Tm should be expressed in kelvins. The equation is a linear approximation of radiative heat transfer for gray, diffuse surfaces and moderate temperature differences.

where:

$$L_{r.STL} = g_r * D_h \quad \text{(S152)}$$

or alternatively [27,34]:

$$L_{r.STL} = g_r * L_c \quad \text{(S153)}$$

For highly open structures: $g_r = 0.5 \ ... \ 1.0$

whereas for strongly branched structure: $g_r = 0.2 \ ... \ 0.5$

<u>Final STL-Based Open-Cell Model</u>

$$k_{eff,STL}^{op} = k_{cond,s} + k_{gas} + k_{conv,eff} + k_{rad,STL} \quad \text{(S154)}$$

where:

$$k_{cond.s} = k_s * \left[\left(\frac{1}{3}\right) * \rho_r + \left(\frac{2}{3}\right) * \rho_r^{\frac{3}{2}}\right] \quad \text{(S155)}$$

$$k_{gas+conv} = Nu_{eff}\left(\frac{\varepsilon}{\tau_g}\right)k_g \quad \text{(S156)}$$

$$k_{conv,eff} = (Nu_{eff} - 1)\left(\frac{\varepsilon}{\tau_g}\right)k_g \quad \text{(S157)}$$

$$k_{rad} = 4\varepsilon_{rad}\sigma T_m^3 L_r \quad \text{(S158)}$$

After substitution of all contributions, the final form becomes:

$$k_{eff,STL}^{op} = k_s[\left(\frac{1}{3}\right)\rho_r + \left(\frac{2}{3}\right)\rho_r^{\frac{3}{2}}] + Nu_{eff}\left(\frac{\varepsilon}{\tau_g}\right)k_g + 4\varepsilon_{rad}\sigma T_m^3 L_{r,STL} \quad \text{(S159)}$$

Final topology-dependent heat-transfer model for open-cell thermal metamaterials:

$$k_{eff} = (1-\varepsilon)^n k_s + \left(\frac{\varepsilon}{\tau_g}\right)k_g + 16\sigma T_{m_r^3 L} + \left(\frac{\varepsilon}{\tau_g}\right)\left(Nu_{eff} - 1\right)k_g \quad \text{(S160)}$$

If we use radiative emissivity:

$$k_{eff} = (1-\varepsilon)^n k_s + \left(\frac{\varepsilon}{\tau_g}\right)k_g + 4\varepsilon_{(rad)\sigma T_{m_r^3 L}} + \left(\frac{\varepsilon}{\tau_g}\right)\left(Nu_{eff} - 1\right)k_g \quad \text{(S161)}$$

References

1. Gibson, L.J. Cellular Solids. *MRS Bull.* **2003**, *28*, 270–274, doi:10.1557/mrs2003.79.
2. Deshpande, V.S.; Ashby, M.F.; Fleck, N.A. Foam Topology: Bending versus Stretching Dominated Architectures. *Acta Mater.* **2001**, *49*, 1035–1040, doi:10.1016/S1359-6454(00)00379-7.
3. Dixit, T.; Al-Hajri, E.; Paul, M.C.; Nithiarasu, P.; Kumar, S. High Performance, Microarchitected, Compact Heat Exchanger Enabled by 3D Printing. *Appl. Therm. Eng.* **2022**, *210*, 118339, doi:10.1016/j.applthermaleng.2022.118339.
4. Siddique, S.H.; Hazell, P.J.; Wang, H.; Escobedo, J.P.; Ameri, A.A.H. Lessons from Nature: 3D Printed Bio-Inspired Porous Structures for Impact Energy Absorption – A Review. *Addit. Manuf.* **2022**, *58*, 103051, doi:10.1016/j.addma.2022.103051.
5. Chen, F.; Jiang, X.; Lu, C.; Wang, Y.; Wen, P.; Shen, Q. Heat Transfer Efficiency Enhancement of Gyroid Heat Exchanger Based on Multidimensional Gradient Structure Design. *International Communications in Heat and Mass Transfer* **2023**, *149*, 107127, doi:10.1016/j.icheatmasstransfer.2023.107127.
6. Bensoussan, A.; Lions, J.-L.; Papanicolaou, G. *Asymptotic Analysis for Periodic Structures*; North-Holland Publishing Company: Amsterdam, The Netherlands, 1978.
7. Sanchez-Palencia, E. *Non-Homogeneous Media and Vibration Theory;* Springer: Berlin, Germany, 1980; Volume 127; ISBN 978-3-540-10000-3.
8. Evans, L.C. *Partial Differential Equations*, 3rd ed.; American Mathematical Society: Providence, RI, USA, 2022; ISBN 978-1470469429..
9. Gibson, I.; Rosen, D.W.; Stucker, B. *Additive Manufacturing Technologies*, 2nd ed.; Springer: New York, NY, USA, 2015; ISBN 978-1-4939-2112-6..
10. Wong, M.; Owen, I.; Sutcliffe, C.J. Pressure Loss and Heat Transfer Through Heat Sinks Produced by Selective Laser Melting. *Heat Transfer Engineering* **2009**, *30*, 1068–1076, doi:10.1080/01457630902922228.
11. Grabowska, B.; Kasperski, J. The Thermal Conductivity of 3D Printed Plastic Insulation Materials—The Effect of Optimizing the Regular Structure of Closures. *Materials* **2020**, *13*, 4400, doi:10.3390/ma13194400.
12. Ashby, M.F.; Evans, A.G.; Fleck, N.A.; Gibson, L.J.; Hutchinson, J.W.; Wadley, H.N.G. *Metal Foams: A Design Guide*; Butterworth-Heinemann: Oxford, UK, 2009.
13. Whitaker, S. *The Method of Volume Averaging*; Kluwer Academic Publishers: Dordrecht, The Netherlands, 1999.
14. Patankar, S. V.; Liu, C.H.; Sparrow, E.M. Fully Developed Flow and Heat Transfer in Ducts Having Streamwise-Periodic Variations of Cross-Sectional Area. *J. Heat Transfer* **1977**, *99*, 180–186, doi:10.1115/1.3450666.
15. Murthy, J.Y.; Mathur, S. Periodic Flow and Heat Transfer Using Unstructured Meshes. *Int. J. Numer. Methods Fluids* **1997**, *25*, 659–677, doi:10.1002/(SICI)1097-0363(19970930)25:6<659::AID-FLD580>3.0.CO;2-Y.
16. Hashin, Z.; Shtrikman, S. A Variational Approach to the Theory of the Elastic Behaviour of Multiphase Materials. *J. Mech. Phys. Solids* **1963**, *11*, 127–140, doi:10.1016/0022-5096(63)90060-7.
17. Lu, T.J.; Stone, H.A.; Ashby, M.F. Heat Transfer in Open-Cell Metal Foams. *Acta Mater.* **1998**, *46*, 3619–3635, doi:10.1016/S1359-6454(98)00031-7.
18. Ashby, M.F. The properties of foams and lattices. *Philos. Trans. R. Soc. A Math. Phys. Eng. Sci.* **2006**, *364*, 15–30, https://doi.org/10.1098/rsta.2005.1678.
19. Calmidi, V. V.; Mahajan, R.L. The Effective Thermal Conductivity of High Porosity Fibrous Metal Foams. *J. Heat Transfer* **1999**, *121*, 466–471, doi:10.1115/1.2826001.

20. Lemlich, R. A Theory for the Limiting Conductivity of Polyhedral Foam at Low Density. *J. Colloid Interface Sci.* **1978**, *64*, 107–110, doi:10.1016/0021-9797(78)90339-9.
21. Paek, J.W.; Kang, B.H.; Kim, S.Y.; Hyun, J.M. Effective thermal conductivity and permeability of aluminum foam materials. *Int. J. Thermophys.* **2000**, *21*, 453–464 doi:10.1023/A:1006643815323.
22. Bhattacharya, A.; Calmidi, V.V.; Mahajan, R.L. Thermophysical Properties of High Porosity Metal Foams. *Int. J. Heat Mass Transf.* **2002**, *45*, 1017–1031, doi:10.1016/S0017-9310(01)00220-4.
23. Chhabra, V.; Bambery, K.; Bhattacharya, S.; Shastri, Y. Thermal and in Situ Infrared Analysis to Characterise the Slow Pyrolysis of Mixed Municipal Solid Waste (MSW) and Its Components. *Renew. Energy* **2020**, *148*, 388–401, doi:10.1016/j.renene.2019.10.045.
24. Wang, Y.; Wang, J.; Jia, P. Performance of Forced Convection Heat Transfer in Porous Media Based on Gibson–Ashby Constitutive Model. *Heat Transfer Engineering* **2011**, *32*, 1093–1098, doi:10.1080/01457632.2011.556508.
25. *Öchsner, A.; Murch, G.E.; de Lemos, M.J.S., Eds. Cellular and Porous Materials in Structures and Processes; Wiley-VCH: Weinheim, Germany, 2008; ISBN 978-3527319381*.
26. Paek, S.W.; Balasubramanian, S.; Stupples, D. Composites Additive Manufacturing for Space Applications: A Review. *Materials* **2022**, *15*, 4709, doi:10.3390/ma15134709.
27. Howell, J.R.; Mengüç, M.P.; Siegel, R. *Thermal Radiation Heat Transfer*, 5th ed.; CRC Press: Boca Raton, FL, USA, 2010; ISBN 978-1439894552.
28. Bergman, T.L.; Lavine, A.S.; Incropera, F.P.; DeWitt, D.P. *Fundamentals of Heat and Mass Transfer*, 7th ed.; John Wiley & Sons: Hoboken, NJ, USA, 2011;
29. Nield, D.A.; Bejan, A. *Convection in Porous Media*, 5th ed.; Springer: Cham, Switzerland, 2017.
30. Bejan, A. *Convection Heat Transfer*, 4th ed.; Wiley: Hoboken, NJ, USA, 2013.
31. Calmidi, V. V.; Mahajan, R.L. Forced Convection in High Porosity Metal Foams. *J. Heat Transfer* **2000**, *122*, 557–565, doi:10.1115/1.1287793.
32. Ranut, P.; Nobile, E. On the Effective Thermal Conductivity of Metal Foams. *J. Phys. Conf. Ser.* **2014**, *547*, 012021, doi:10.1088/1742-6596/547/1/012021.
33. Benveniste, Y. On the effective thermal conductivity of multiphase composites. *Z. Angew. Math. Phys.* **1986**, *37*, 696–713. https://doi.org/10.1007/BF00947917.
34. Kaviany, M. *Principles of Heat Transfer in Porous Media*, 2nd ed.; Springer: New York, NY, USA, 1995..

Table S1. Comparison of the mathematical-model predictions for the investigated lattice structures.

Structure	Porosity	Heat-Transfer Direction

	L_c [mm]	[%]	Top-to-Bottom Heat Transfer	Bottom-to-Top Heat Transfer
			k_{eff} [W/m·K]	k_{eff} [W/m·K]
**All_face_cubic**	4	50	0.108	0.112
		70	0.073	0.076
		90	0.046	0.049
		95	0.041	0.043
	5	50	0.110	0.114
		70	0.075	0.078
		90	0.048	0.051
		95	0.043	0.045
	6	50	0.112	0.116
		70	0.077	0.080
		90	0.050	0.053
		95	0.045	0.048
	7	50	0.114	0.118
		70	0.080	0.083
		90	0.053	0.055
		95	0.048	0.050
	8	50	0.117	0.120
		70	0.082	0.085
		90	0.055	0.057
		95	0.050	0.052
	9	50	0.119	0.122
		70	0.084	0.087
		90	0.057	0.059
		95	0.052	0.054
	10	50	0.121	0.124
		70	0.086	0.089
		90	0.059	0.061
		95	0.054	0.056
**Auxetic**	4	50	0.103	0.112
		70	0.070	0.076
		90	0.044	0.049
		95	0.039	0.043
	5	50	0.105	0.114
		70	0.072	0.078
		90	0.046	0.051
		95	0.041	0.045
	6	50	0.108	0.116
		70	0.074	0.080
		90	0.048	0.053
		95	0.043	0.048
	7	50	0.110	0.118
		70	0.076	0.083

		90	0.050	0.055
		95	0.046	0.050
	8	50	0.112	0.120
		70	0.078	0.085
		90	0.052	0.057
		95	0.048	0.052
	9	50	0.114	0.122
		70	0.080	0.087
		90	0.055	0.059
		95	0.050	0.054
	10	50	0.116	0.124
		70	0.083	0.089
		90	0.057	0.061
		95	0.052	0.056
**BCC**	4	50	0.099	0.112
		70	0.067	0.076
		90	0.042	0.049
		95	0.038	0.043
	5	50	0.101	0.114
		70	0.069	0.078
		90	0.044	0.051
		95	0.040	0.045
	6	50	0.103	0.116
		70	0.071	0.080
		90	0.046	0.053
		95	0.041	0.048
	7	50	0.105	0.118
		70	0.073	0.083
		90	0.048	0.055
		95	0.043	0.050
	8	50	0.107	0.120
		70	0.075	0.085
		90	0.050	0.057
		95	0.045	0.052
	9	50	0.109	0.122
		70	0.077	0.087
		90	0.052	0.059
		95	0.047	0.054
	10	50	0.111	0.124
		70	0.079	0.089
		90	0.054	0.061
		95	0.049	0.056
**BCCZ**	4	50	0.099	0.112
		70	0.067	0.076
		90	0.042	0.049
		95	0.038	0.043

	5	50	0.101	0.114
		70	0.069	0.078
		90	0.044	0.051
		95	0.040	0.045
	6	50	0.103	0.116
		70	0.071	0.080
		90	0.046	0.053
		95	0.042	0.048
	7	50	0.105	0.118
		70	0.073	0.083
		90	0.048	0.055
		95	0.044	0.050
	8	50	0.107	0.120
		70	0.075	0.085
		90	0.050	0.057
		95	0.046	0.052
	9	50	0.109	0.122
		70	0.077	0.087
		90	0.052	0.059
		95	0.048	0.054
	10	50	0.111	0.124
		70	0.079	0.089
		90	0.580	0.061
		95	0.050	0.056
**CUBIC**	4	50	0.104	0.112
		70	0.071	0.076
		90	0.044	0.049
		95	0.040	0.043
	5	50	0.106	0.114
		70	0.073	0.078
		90	0.047	0.051
		95	0.042	0.045
	6	50	0.108	0.116
		70	0.075	0.080
		90	0.049	0.053
		95	0.044	0.048
	7	50	0.111	0.118
		70	0.077	0.083
		90	0.051	0.055
		95	0.046	0.050
	8	50	0.113	0.120
		70	0.079	0.085
		90	0.053	0.057
		95	0.048	0.052
	9	50	0.115	0.122
		70	0.081	0.087

		90	0.055	0.059
		95	0.050	0.054
	10	50	0.117	0.124
		70	0.083	0.089
		90	0.057	0.061
		95	0.052	0.056
**Cuboctahedron Z**	4	50	0.109	0.112
		70	0.074	0.076
		90	0.047	0.049
		95	0.042	0.043
	5	50	0.112	0.114
		70	0.076	0.078
		90	0.049	0.051
		95	0.044	0.045
	6	50	0.114	0.116
		70	0.078	0.080
		90	0.051	0.053
		95	0.046	0.048
	7	50	0.116	0.118
		70	0.081	0.083
		90	0.053	0.055
		95	0.048	0.050
	8	50	0.118	0.120
		70	0.083	0.085
		90	0.055	0.057
		95	0.050	0.052
	9	50	0.120	0.122
		70	0.085	0.087
		90	0.058	0.059
		95	0.053	0.054
	10	50	0.123	0.124
		70	0.087	0.089
		90	0.060	0.061
		95	0.055	0.056
**Diamond**	4	50	0.101	0.112
		70	0.068	0.076
		90	0.043	0.049
		95	0.038	0.043
	5	50	0.103	0.114
		70	0.070	0.078
		90	0.045	0.051
		95	0.040	0.045
	6	50	0.105	0.116
		70	0.072	0.080
		90	0.047	0.053
		95	0.042	0.048

	7	50	0.107	0.118
		70	0.074	0.083
		90	0.049	0.055
		95	0.044	0.050
	8	50	0.109	0.120
		70	0.076	0.085
		90	0.051	0.057
		95	0.046	0.052
	9	50	0.111	0.122
		70	0.078	0.087
		90	0.053	0.059
		95	0.048	0.054
	10	50	0.113	0.124
		70	0.080	0.089
		90	0.055	0.061
		95	0.051	0.056
**FBCC**	4	50	0.107	0.112
		70	0.072	0.076
		90	0.046	0.049
		95	0.041	0.043
	5	50	0.109	0.114
		70	0.075	0.078
		90	0.048	0.051
		95	0.043	0.045
	6	50	0.111	0.116
		70	0.077	0.080
		90	0.050	0.053
		95	0.045	0.048
	7	50	0.113	0.118
		70	0.079	0.083
		90	0.052	0.055
		95	0.047	0.050
	8	50	0.116	0.120
		70	0.081	0.085
		90	0.054	0.057
		95	0.049	0.052
	9	50	0.118	0.122
		70	0.083	0.087
		90	0.056	0.059
		95	0.051	0.054
	10	50	0.120	0.124
		70	0.085	0.089
		90	0.059	0.061
		95	0.054	0.056
**FBCCXYZ**	4	50	0.123	0.112
		70	0.083	0.076

		90	0.052	0.049
		95	0.047	0.043
	5	50	0.125	0.114
		70	0.086	0.078
		90	0.055	0.051
		95	0.049	0.045
	6	50	0.128	0.116
		70	0.088	0.080
		90	0.057	0.053
		95	0.052	0.048
	7	50	0.130	0.118
		70	0.091	0.083
		90	0.060	0.055
		95	0.054	0.050
	8	50	0.133	0.120
		70	0.093	0.085
		90	0.062	0.057
		95	0.057	0.052
	9	50	0.135	0.122
		70	0.096	0.087
		90	0.065	0.059
		95	0.059	0.054
	10	50	0.138	0.124
		70	0.098	0.089
		90	0.067	0.061
		95	0.062	0.056
**FBCCZ**	4	50	0.112	0.112
		70	0.076	0.076
		90	0.048	0.049
		95	0.043	0.043
	5	50	0.115	0.114
		70	0.078	0.078
		90	0.050	0.051
		95	0.045	0.045
	6	50	0.117	0.116
		70	0.081	0.080
		90	0.052	0.053
		95	0.047	0.048
	7	50	0.119	0.118
		70	0.083	0.083
		90	0.055	0.055
		95	0.050	0.050
	8	50	0.121	0.120
		70	0.085	0.085
		90	0.057	0.057
		95	0.052	0.052

	9	50	0.124	0.122
		70	0.087	0.087
		90	0.059	0.059
		95	0.054	0.054
	10	50	0.126	0.124
		70	0.090	0.089
		90	0.062	0.061
		95	0.056	0.056
**FCC**	4	50	0.103	0.112
		70	0.070	0.076
		90	0.044	0.049
		95	0.039	0.043
	5	50	0.105	0.114
		70	0.072	0.078
		90	0.046	0.051
		95	0.041	0.045
	6	50	0.107	0.116
		70	0.074	0.080
		90	0.048	0.053
		95	0.043	0.048
	7	50	0.109	0.118
		70	0.076	0.083
		90	0.050	0.055
		95	0.045	0.050
	8	50	0.111	0.120
		70	0.078	0.085
		90	0.052	0.057
		95	0.047	0.052
	9	50	0.113	0.122
		70	0.080	0.087
		90	0.054	0.059
		95	0.049	0.054
	10	50	0.115	0.124
		70	0.082	0.089
		90	0.056	0.061
		95	0.051	0.056
**FCCZ**	4	50	0.104	0.112
		70	0.071	0.076
		90	0.045	0.049
		95	0.040	0.043
	5	50	0.107	0.114
		70	0.073	0.078
		90	0.047	0.051
		95	0.042	0.045
	6	50	0.109	0.116
		70	0.075	0.080

		90	0.049	0.053
		95	0.044	0.048
	7	50	0.111	0.118
		70	0.077	0.083
		90	0.051	0.055
		95	0.046	0.050
	8	50	0.113	0.120
		70	0.079	0.085
		90	0.053	0.057
		95	0.048	0.052
	9	50	0.115	0.122
		70	0.081	0.087
		90	0.055	0.059
		95	0.050	0.054
	10	50	0.117	0.124
		70	0.083	0.089
		90	0.057	0.061
		95	0.052	0.056
**G7_PANEL**	4	50	0.107	0.112
		70	0.072	0.076
		90	0.046	0.049
		95	0.041	0.043
	5	50	0.109	0.114
		70	0.074	0.078
		90	0.048	0.051
		95	0.043	0.045
	6	50	0.111	0.116
		70	0.077	0.080
		90	0.050	0.053
		95	0.045	0.048
	7	50	0.113	0.118
		70	0.079	0.083
		90	0.052	0.055
		95	0.047	0.050
	8	50	0.115	0.120
		70	0.081	0.085
		90	0.054	0.057
		95	0.049	0.052
	9	50	0.117	0.122
		70	0.083	0.087
		90	0.056	0.059
		95	0.051	0.054
	10	50	0.120	0.124
		70	0.085	0.089
		90	0.058	0.061
		95	0.053	0.056

Iso truss	4	50	0.114	0.112
		70	0.077	0.076
		90	0.049	0.049
		95	0.043	0.043
	5	50	0.116	0.114
		70	0.080	0.078
		90	0.051	0.051
		95	0.046	0.045
	6	50	0.119	0.116
		70	0.082	0.080
		90	0.053	0.053
		95	0.048	0.048
	7	50	0.121	0.118
		70	0.084	0.083
		90	0.056	0.055
		95	0.050	0.050
	8	50	0.123	0.120
		70	0.087	0.085
		90	0.058	0.057
		95	0.053	0.052
	9	50	0.126	0.122
		70	0.089	0.087
		90	0.060	0.059
		95	0.055	0.054
	10	50	0.128	0.124
		70	0.091	0.089
		90	0.062	0.061
		95	0.057	0.056
Kelvin	4	50	0.105	0.112
		70	0.071	0.076
		90	0.045	0.049
		95	0.040	0.043
	5	50	0.107	0.114
		70	0.073	0.078
		90	0.047	0.051
		95	0.042	0.045
	6	50	0.109	0.116
		70	0.075	0.080
		90	0.049	0.053
		95	0.044	0.048
	7	50	0.111	0.118
		70	0.077	0.083
		90	0.051	0.055
		95	0.046	0.050
	8	50	0.113	0.120
		70	0.079	0.085

		90	0.053	0.057
		95	0.048	0.052
	9	50	0.115	0.122
		70	0.081	0.087
		90	0.055	0.059
		95	0.050	0.054
	10	50	0.117	0.124
		70	0.083	0.089
		90	0.057	0.061
		95	0.052	0.056
**Octahedron**	4	50	0.101	0.112
		70	0.068	0.076
		90	0.043	0.049
		95	0.038	0.043
	5	50	0.103	0.114
		70	0.070	0.078
		90	0.045	0.051
		95	0.040	0.045
	6	50	0.105	0.116
		70	0.072	0.080
		90	0.047	0.053
		95	0.042	0.048
	7	50	0.107	0.118
		70	0.074	0.083
		90	0.049	0.055
		95	0.044	0.050
	8	50	0.109	0.120
		70	0.076	0.085
		90	0.051	0.057
		95	0.046	0.052
	9	50	0.111	0.122
		70	0.078	0.087
		90	0.053	0.059
		95	0.048	0.054
	10	50	0.113	0.124
		70	0.080	0.089
		90	0.055	0.061
		95	0.050	0.056
**Octet**	4	50	0.115	0.112
		70	0.078	0.076
		90	0.049	0.049
		95	0.044	0.043
	5	50	0.117	0.114
		70	0.080	0.078
		90	0.051	0.051
		95	0.046	0.045

	6	50	0.120	0.116
		70	0.083	0.080
		90	0.054	0.053
		95	0.048	0.048
	7	50	0.122	0.118
		70	0.085	0.083
		90	0.056	0.055
		95	0.051	0.050
	8	50	0.124	0.120
		70	0.087	0.085
		90	0.058	0.057
		95	0.053	0.052
	9	50	0.127	0.122
		70	0.090	0.087
		90	0.061	0.059
		95	0.055	0.054
	10	50	0.129	0.124
		70	0.092	0.089
		90	0.063	0.061
		95	0.058	0.056
**Rhombic dodecahedron**	4	50	0.107	0.112
		70	0.072	0.076
		90	0.046	0.049
		95	0.041	0.043
	5	50	0.109	0.114
		70	0.074	0.078
		90	0.048	0.051
		95	0.043	0.045
	6	50	0.111	0.116
		70	0.077	0.080
		90	0.050	0.053
		95	0.045	0.048
	7	50	0.113	0.118
		70	0.079	0.083
		90	0.052	0.055
		95	0.047	0.050
	8	50	0.115	0.120
		70	0.081	0.085
		90	0.054	0.057
		95	0.049	0.052
	9	50	0.117	0.122
		70	0.083	0.087
		90	0.056	0.059
		95	0.051	0.054
	10	50	0.119	0.124
		70	0.085	0.089

		90	0.058	0.061
		95	0.053	0.056
Tetrahedron	4	50	0.137	0.112
		70	0.092	0.076
		90	0.058	0.049
		95	0.052	0.043
	5	50	0.139	0.114
		70	0.095	0.078
		90	0.061	0.051
		95	0.055	0.045
	6	50	0.142	0.116
		70	0.098	0.080
		90	0.064	0.053
		95	0.057	0.048
	7	50	0.145	0.118
		70	0.101	0.083
		90	0.066	0.055
		95	0.060	0.050
	8	50	0.147	0.120
		70	0.103	0.085
		90	0.069	0.057
		95	0.063	0.052
	9	50	0.150	0.122
		70	0.106	0.087
		90	0.072	0.059
		95	0.066	0.054
	10	50	0.153	0.124
		70	0.109	0.089
		90	0.075	0.061
		95	0.068	0.056
Truncated cube	4	50	0.107	0.112
		70	0.072	0.076
		90	0.046	0.049
		95	0.041	0.043
	5	50	0.109	0.114
		70	0.074	0.078
		90	0.048	0.051
		95	0.043	0.045
	6	50	0.111	0.116
		70	0.077	0.080
		90	0.050	0.053
		95	0.045	0.048
	7	50	0.113	0.118
		70	0.079	0.083
		90	0.052	0.055
		95	0.047	0.050

	8	50	0.115	0.120
		70	0.081	0.085
		90	0.054	0.057
		95	0.049	0.052
	9	50	0.117	0.122
		70	0.083	0.087
		90	0.056	0.059
		95	0.051	0.054
	10	50	0.120	0.124
		70	0.085	0.089
		90	0.058	0.061
		95	0.053	0.056
**Truncated cuboctahedron**	4	50	0.112	0.112
		70	0.076	0.076
		90	0.048	0.049
		95	0.043	0.043
	5	50	0.114	0.114
		70	0.078	0.078
		90	0.050	0.051
		95	0.045	0.045
	6	50	0.116	0.116
		70	0.080	0.080
		90	0.052	0.053
		95	0.047	0.048
	7	50	0.119	0.118
		70	0.083	0.083
		90	0.054	0.055
		95	0.049	0.050
	8	50	0.121	0.120
		70	0.085	0.085
		90	0.057	0.057
		95	0.052	0.052
	9	50	0.123	0.122
		70	0.087	0.087
		90	0.059	0.059
		95	0.054	0.054
	10	50	0.125	0.124
		70	0.089	0.089
		90	0.061	0.061
		95	0.056	0.056
